# A Shared Threshold Rule for Ants and Workers: Multi-Objective Design of Labour-Force Participation Policy and a Division-of-Labour Evolutionary Algorithm

**DOI:** 10.3390/biomimetics11100766

**Published:** 2026-10-21

**Authors:** Ge Liang, Xinyu Cai

**Affiliations:** 1 School of Labor Economics, Capital University of Economics and Business, Beijing 100070, China; 12021050012@cueb.edu.cn; 2 College of Business, Jiaxing University, Jiaxing 314001, China

**Keywords:** division of labour, response threshold, multi-objective evolutionary algorithm, labour force participation, tax-benefit design

## Abstract

Tax-benefit policy acts most directly on whether adults work. We show that the response-threshold rule by which social insects divide labour and the extensive-margin participation curve share one functional form when the private cost of working is additive and log-logistic; an assumed participation elasticity then implies the exponent. The correspondence defines a tax-benefit design problem with three objectives—participation, welfare and fiscal cost–whose exact supported Pareto front we compute, and an eleven-instrument version for single adults and lone parents. It also drives MOEA/D-DoL, a decomposition-based algorithm whose subproblems are served by threshold-driven workers that compare their success with the colony’s. By IGD, MOEA/D-DoL beats its MOEA/D-DE core clearly on two of seventeen benchmarks, both used in its design, and on two of nine held-out WFG problems, losing on one benchmark; AR-MOEA, NSGA-III and NSGA-II rank higher on both suites. The schedules that raise participation most at no extra cost lower the guaranteed income and pay an in-work credit financed by a higher tax rate. This direction survives joint calibration draws, a 100% cap on marginal rates and wider instrument ranges, but the tax is cheap partly because hours do not respond, and the gain varies in size.

## 1. Introduction

Whether an adult works at all is the margin on which much of labour-market policy operates. Participation has been a central margin of the long-run movements in employment in the United States, above all for women [1,2], whose participation has since fallen behind that of women in other rich countries [3]. In Denmark, the arrival of children opens a long-run gender gap in earnings that runs through participation and hours as well as wage rates [4]. In China, the labour-force participation of married women in cities fell through the two decades after 1991 [5], and the gender gap in participation has widened partly because women are required to retire earlier than men [6]. Each of these is a statement about the extensive margin, i.e., about who decides that working is worth its cost.

Tax-benefit systems act on that decision directly. The participation tax rate, the share of earnings lost to taxes and withdrawn benefits when a person moves from non-work into work, is high in many countries: around 80 per cent in Scandinavia [7], and in most countries of the pre-2004 European Union, the large existing welfare programmes with high phase-out rates make in-work benefits a more desirable reform than more generous traditional welfare [8]. The evidence on what such instruments achieve is real but contested. The expansions of the Earned Income Tax Credit raised the participation of single mothers [9,10] while lowering that of married women [11], and a reappraisal finds much less of an extensive-margin effect than the earlier literature [12]. Subsidised childcare raised maternal labour supply in Quebec [13,14] but not in Norway [15], and sixty years of Austrian family policy reform left gender convergence virtually untouched [16]. Across countries, the extensive margin dominates the intensive one, and among single people, the response is larger in low-income groups [17]; in the United Kingdom, labour supply is particularly elastic among lone mothers [18].

Optimal-tax theory, built on the model of Mirrlees [19], says what an ideal schedule should look like once participation responds. When the extensive margin matters, the classic intensive-margin result that marginal tax rates are never negative can reverse at the bottom, and a negative participation tax, an earned income credit, can be optimal [20,21,22,23,24]. Those results characterise a schedule that is free to take any value at every income. A real schedule is assembled from a handful of instruments, a guaranteed income, a benefit taper, an earnings disregard, a tax allowance, a credit with a phase-in, a plateau and a phase-out, which interact through kinks and cliffs and apply at once to households of different types. A planner choosing their values faces three goals that pull apart: more people in work, higher social welfare, and a lower net cost to the public purse. That is a multi-objective search over a non-smooth space. Evolutionary methods have already been used for personal income-tax reform, where a genetic algorithm searched a large combinatorial space of tax structures for the most redistributive one at a given revenue [25].

Among multi-objective evolutionary algorithms, the decomposition framework MOEA/D replaces the search for a Pareto front by a set of scalar subproblems solved in cooperation [26,27], and the allocation of computational effort among those subproblems is one of the directions in which it has been developed [28]. Treating all subproblems equally is the naive choice; allocating effort according to a measured utility of each subproblem improves on it [29], and the variation operators themselves can be chosen adaptively by a multi-armed bandit [30]. These rules are engineered for the purpose, each with its own measure of utility, update period and thresholds.

Social insects face the same allocation problem, with a rule that nobody designed for it. The division of labour of a colony is plastic, workers changing tasks as conditions change [31], and self-organisation models explain this without assuming complex cognition or central control [32]. In the response-threshold model, a worker engages in a task with high probability once its stimulus exceeds her threshold [33]. Thresholds need not be fixed: if performing a task lowers the threshold for it and idleness raises it, experience alone turns a homogeneous group into specialists [34], as it does in ants [35]. The threshold rule has allocated tasks in groups of robots [36]; social-insect behaviour more broadly has inspired optimisation algorithms [37]; and the rule itself has recently been used to divide exploration and exploitation between individuals of a single-objective swarm [38]. As nature-inspired algorithms multiply, how they are compared matters as much as what inspires them: their rankings can change with the evaluation protocol, for instance with the evaluation budget [39]. A borrowed mechanism is therefore most convincing when it is stated precisely, removed in an ablation and tested on problems that played no part in its design, which is what Section 4 and Section 5 do.

This paper starts from an observation that links the two halves of the problem. The response-threshold function of the social insects and the participation curve of labour economics can share one functional form. An adult whose private cost of working is drawn from a log-logistic distribution works with exactly the probability with which an insect worker takes up a task in the fixed-threshold model, with the utility gain from work as the stimulus and the scale of the cost distribution as the threshold. This holds when only the extensive margin responds and the private cost of working enters utility additively, does not depend on consumption or on the schedule, and is log-logistic (Section 2.2); other S-shaped cost distributions reproduce the threshold form only approximately. Under these assumptions the colony and the labour market allocate participation by the same rule. We borrow from it in two different ways. In the policy problem, the borrowing is quantitative: the response function, with an exponent implied by assumed participation elasticities, describes how adults react to a schedule. In the algorithm, only the mechanism is borrowed: every subproblem is served by a worker that engages with the probability of the response function, whose stimulus evolves as in the colony models and whose threshold follows a version of threshold reinforcement that measures success against the colony’s own rate, while the exponent is a design constant, *n* = 2, that no labour-market estimate fixes; setting it to the labour-market values changes little (Section 5.5).

The contributions are the following.

1. A formal correspondence between the fixed-threshold model of division of labour and extensive-margin labour supply (Proposition 1), with the elasticity identity that ties the insect response exponent to the participation elasticity, so that an assumed or estimated elasticity implies its value.
2. MOEA/D-DoL, a decomposition-based algorithm in which every subproblem is a task and every individual a worker who engages with probability given by the response function; stimuli accumulate while a task waits and are consumed by work, and thresholds fall for workers that succeed more often than the colony and rise for those that succeed less often, after the ant experiment of Ravary et al. We show that a literal transfer of the reinforcement rule dissolves the division of labour in an optimiser and that the relative version keeps it alive. On seventeen benchmark problems, MOEA/D-DoL is significantly better by IGD than the MOEA/D-DE core it is built on in three problems and worse in one; its two clear gains are on problems used in its design; the third is negligible (the medians differ by less than 1%), and on nine held-out WFG problems, it is better than its core on three, by 9.5% and 1.1% in median IGD on two of them and negligibly on the third, and worse on none.
3. A participation-oriented tax-benefit design problem whose supported Pareto front can be computed exactly, and in which an ε-constraint check finds nothing essential outside that front, so that the algorithms are scored against the truth rather than against each other. On it, the most accurate algorithm is AR-MOEA, which normalises the objectives, and every decomposition algorithm, MOEA/D-DoL included, covers only about a quarter of the front; normalising the objectives inside the decomposition closes most of the IGD gap and doubles the coverage, as the original MOEA/D paper anticipated for disparately scaled objectives [26].
4. A realistic instrument-based version for single adults and lone parents, and what its Pareto set says about in-work credits, benefit tapers and childcare support. At no extra cost, the Pareto-optimal schedule that raises participation most lowers the guaranteed income and pays an in-work credit financed by a higher income-tax rate. This holds in every single-parameter and joint calibration we tried, and also, when marginal rates are capped at 100% or the instrument ranges are widened, although the low cost of that tax rate partly reflects the absence of an hours margin. The guaranteed income out of work decides how participation is traded against welfare, and the size of the gain depends on the calibration and on the ranges allowed to the instruments.

Section 2 sets out the response-threshold model and the correspondence. Section 3 formulates the two policy-design problems and the exact solution method. Section 4 describes MOEA/D-DoL. Section 5 evaluates it on standard benchmarks and Section 6 on the policy problems. Section 7 discusses what the biology contributes and where the analysis stops, and Section 8 concludes.

## 2. Response Thresholds in Colonies and in Labour Markets

### 2.1. Division of Labour in Social Insects

The division of labour of a social-insect colony is plastic: workers shift between tasks as the needs of the colony and its environment change [31]. Models of how this happens are based on both internal and external factors [40], and the response-threshold model is a prominent class among the self-organisation models [32]. Each task *j* emits a stimulus *s_j_*, such as the hunger signal of larvae or the temperature of the nest, and each worker *i* has a threshold *θ_ij_* for it. In the fixed-threshold model of Bonabeau, Theraulaz and Deneubourg [33], a worker engages in a task with high probability once its stimulus exceeds her threshold, and successful performance of the task reduces the stimulus. We write the probability of engaging as the response function

(1) $$T_{\theta }(s)=\frac{s^{n}}{s^{n}+\theta^{n}},$$

which rises from zero to one around *s* = *θ* with a steepness set by the exponent *n*, and let the stimulus grow by *δ* in each period and fall in proportion to the share of workers that perform the task,

(2) $$s_{j}(t+1)=s_{j}(t)+\delta -\alpha \frac{N_{j}^{\mathrm{act}}(t)}{N},$$

so that a neglected task becomes more urgent until someone attends to it. Workers with low thresholds respond first; if they cannot meet the demand, the stimulus keeps rising and recruits workers with higher thresholds. Variation in thresholds therefore produces a graded colony response rather than an all-or-nothing one.

Thresholds need not be fixed. In the reinforcement model of Theraulaz, Bonabeau and Deneubourg [34] a worker’s threshold for a task falls by *ξ* for every period in which she performs it and rises by *ϕ* for every period in which she does not, so that experience makes her a specialist and idleness makes her a reluctant generalist. The prediction has been confirmed experimentally: in the ant *Cerapachys biroi*, individual experience alone generated a lasting division of labour among otherwise identical workers [35]. The model is a simplification: task choice in real colonies depends on further factors, and other mechanisms have been proposed alongside it [32,40]. Its simplicity has made it portable: it has allocated tasks among cooperating robots [36] and divided roles in a swarm optimiser [38].

### 2.2. The Labour Market as a Threshold System

Labour economics describes the decision to work in the same terms. An adult works if the wage offered exceeds the value she places on her time [41], and fixed costs of going to work, which differ across people, are of prime importance for whether married women work [42]. Let an adult at earnings level *i* consume *c_i_* if she works and *c*_0_ if she does not, with utility ln *c* − *ϑ* 𝟙[work], where *ϑ* is her private cost of working and the indicator equals one if she works and zero otherwise. She works if and only if *ϑ* ≤ *D_i_*, where

*D_i_* = ln *c_i_* − ln *c*_0_ (3)

is the utility gain from work. The share of adults at level *i* who work is the distribution function of the cost of working evaluated at *D_i_*, and one choice of that distribution turns it into Equation (1).

**Proposition** **1.** *If the cost of working at level i follows a log-logistic distribution with scale a_i_ *> 0* and shape n *> 0*, the participation rate is *$P_{i}=T_{a_{i}}(D_{i})$ *for D_i_* > 0 *and zero otherwise: an adult drawn at random from level i works with exactly the probability with which a worker with threshold a_i_ engages in a task whose stimulus is D_i_. The elasticity of participation with respect to the stimulus is *

(4) ∂ln Pi∂ln Di=n(1−Pi).

**Proof.**  The log-logistic distribution function is $F(\vartheta )=\vartheta^{n}/(\vartheta^{n}+a_{i}^{n})$ for *ϑ* > 0, so $P_{i}=\mathrm{Pr}(\vartheta \leq D_{i})=F(D_{i})=T_{a_{i}}(D_{i})$. Differentiating, $\partial P_{i}/\partial D_{i}=nD_{i}^{n-1}a_{i}^{n}/(D_{i}^{n}+a_{i}^{n}{)}^{2}$, and multiplying by *D_i_*/*P_i_* gives $na_{i}^{n}/(D_{i}^{n}+a_{i}^{n})=n(1-P_{i})$. □

Proposition 1 rests on four assumptions. The decision is binary: an adult at level *i* either works and earns *w_i_* or does not work, so only the extensive margin responds. Utility is additive in log consumption and in the private cost of working, which is incurred only in work. The cost is independent of consumption and of the schedule. And it is log-logistic, with a scale specific to the earnings level and a shape common to all levels of a household type. The first three assumptions make participation equal to the cost distribution evaluated at the utility gain, *P_i_* = *F*(*D_i_*), for any continuous distribution *F*; only the fourth makes *F* the response function (1) exactly. With a log-normal cost, for instance, participation is a probit in ln *D_i_*; calibrated to the same status quo and mean elasticity of the free-schedule economy, it differs from the log-logistic curve by at most 2.0 percentage points for gains between half and twice their status quo values, and by at most 1.2 points on the exact front of Section 3.2 (a Weibull cost differs by up to 5.5 and 3.1 points), but its elasticity is no longer exactly *n*(1 − *P_i_*). The correspondence is therefore exact for the log-logistic family and approximate for similar S-shaped families; it does not cover an hours margin, joint decisions of couples, or costs of working that respond to the schedule.

Optimal-tax theory and the microsimulation work built on it define participation elasticities with respect to the financial gain from work, Δ*_i_* = *c_i_* − *c*_0_, rather than the utility gain [8,21]. Holding *c*_0_ fixed, ∂*D_i_*/∂Δ*_i_* = 1/*c_i_*, which gives the version that can be matched to data.

**Corollary** **1.** *With ρ_i_* = *c_i_*/*c*_0_*, the elasticity of participation with respect to the financial gain from work is*

(5) ηi=∂ln Pi∂ln Δi=n(1−Pi)ρi−1ρiln ρi.

**Figure 1:**
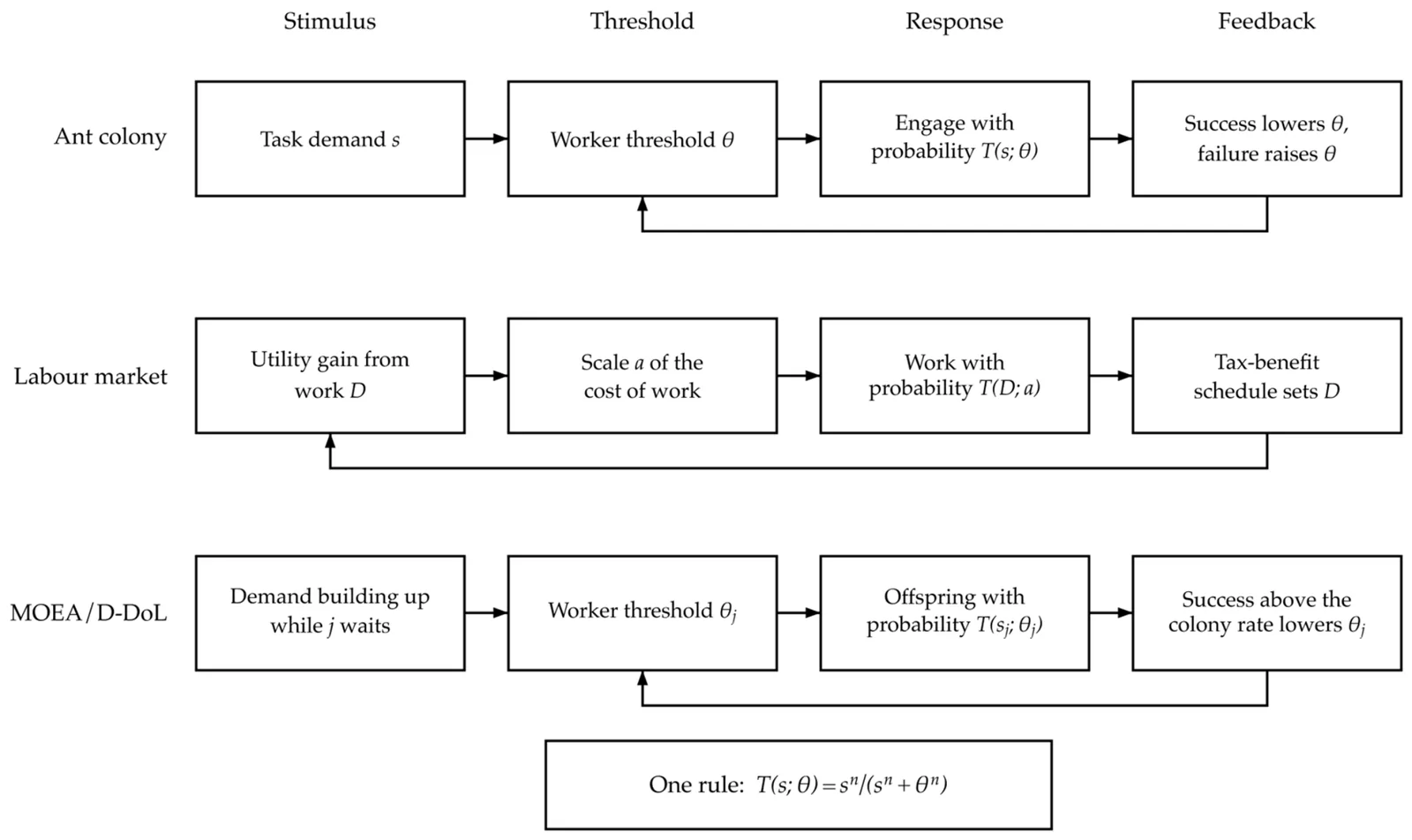
A shared threshold rule in three systems: a colony, a labour market and MOEA/D-DoL.

For *ρ_i_* > 1 the last factor lies between zero and one and tends to one as the gain from work becomes small. Two things follow. First, the response exponent *n* of the insect model is not a free modelling constant in the labour market: given the participation rates and the consumption ratios *ρ_i_*, it is proportional to the participation elasticity. In this paper the elasticities are targets that we set (Section 3.4) rather than estimates, so the exponents inherit the assumptions behind those targets; an estimated elasticity would deliver an estimated exponent through the same identity. Second, the model predicts that participation responds most where participation is lowest, which is consistent with the larger responses that the cross-country evidence finds among low-income single people [17] and with the particularly elastic labour supply of lone mothers [18]. Table 1 collects the correspondence and the third column that Section 4 adds to it, and Figure 1 illustrates it.

**Figure 2:**
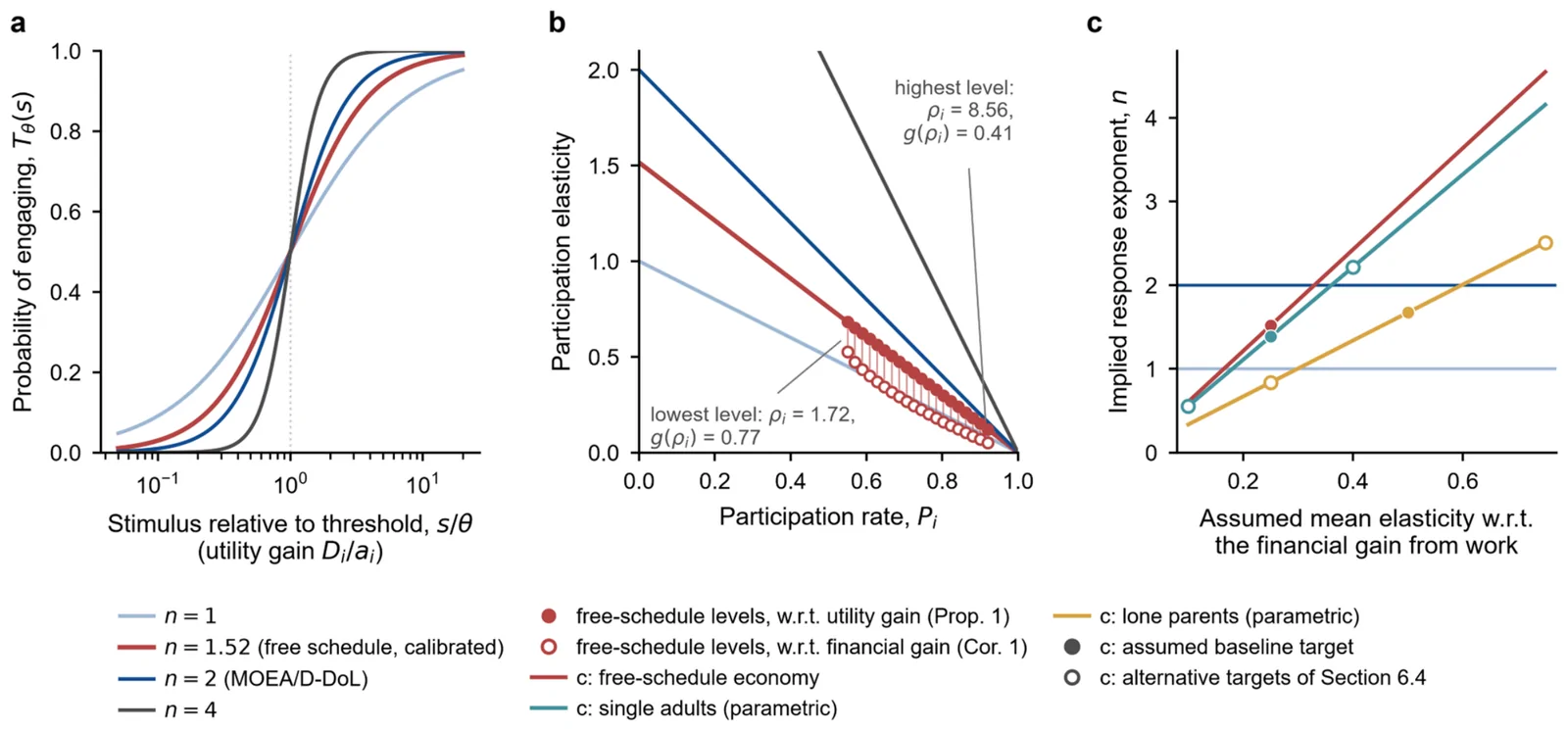
Response threshold function and participation elasticity: (**a**) engagement probability against stimulus; (**b**) elasticity against participation rate; (**c**) implied exponent against assumed elasticity; in (**c**), filled dots mark the baseline targets and open dots the alternative targets of Section 6.4 for single adults and lone parents.

Figure 2 shows what the shape parameter does. A small *n* gives a flat response whose elasticity is low everywhere; a large *n* gives a switch that is highly elastic below and around the threshold and inelastic above it, where almost everyone already takes part. The calibration of Section 3 puts the labour market at *n* = 1.52 in the free-schedule economy, and *n* = 1.38 for single adults and *n* = 1.67 for lone parents in the parametric one: participation responds to the gain from work gradually rather than as a switch, and most elastically where it is lowest. Figure 2b plots each earnings level of the free-schedule economy twice. Its elasticity with respect to the utility gain lies on the line *n*(1 − *P*) of Proposition 1; its elasticity with respect to the financial gain, the quantity that empirical studies report and calibration targets, lies below by the factor (*ρ_i_* − 1)/(*ρ_i_*ln *ρ_i_*) of Corollary 1, which falls from 0.77 at the lowest earnings level to 0.41 at the highest, because a unit of consumption adds less utility where consumption in work is already high. The distance matters for calibration: read as an elasticity with respect to the utility gain, the target of 0.25 would give *n* = 0.94 instead of 1.52. It also matters for policy: the same proportional rise in the cash gain raises participation, in proportional terms, 10.6 times less at the highest earnings level than at the lowest, against 5.6 times for the same proportional rise in the utility gain, and a cash gain of 0.05 raises participation by 5.8 points at the lowest level but by 0.1 point at the highest. Figure 2c shows that the exponent is proportional to the assumed elasticity.

## 3. Participation-Oriented Tax-Benefit Design

### 3.1. The Participation Economy

The economy has *I* earnings levels. A share *ω_i_* of adults can earn *w_i_* if they work, and each draws a private cost of working from the log-logistic distribution of Proposition 1 with scale *a_i_* and a shape *n* common to all levels. The planner sets consumption *c*_0_ for adults who do not work and *c_i_* for those who work at level *i*; the net transfer to a non-worker is *c*_0_ and to a worker *c_i_* − *w_i_*, negative when she pays tax on balance. Three quantities describe an outcome. The participation rate is

(6) $$P=\sum_{i=1}^{I}\omega_{i}T_{a_{i}}(D_{i}),$$

with $T_{a_{i}}(D)=0$ for *D* ≤ 0, as in Proposition 1: nobody works for a loss in consumption. Utilitarian welfare, the expected value of ln *c* − *ϑ* 𝟙[work], takes a closed form after integration by parts,

(7) W=ln c0+∑i=1IωiΦi(Di),  Φi(D)=∫0DTai(t)dt=D−aiz2F11,1n;1+1n;−zn,      z=D/ai,

and *Φ_i_*(*D*) = 0 for *D* ≤ 0, where _2_*F*_1_ is the Gauss hypergeometric function: every non-worker enjoys ln *c*_0_, and every worker enjoys, in addition, the part of her gain *D_i_* that her cost of working does not use up. The net fiscal cost per adult is

(8) $$C=c_{0}+\sum_{i=1}^{I}\omega_{i}T_{a_{i}}(D_{i})(c_{i}-w_{i}-c_{0}).$$

A planner who wants more adults in work, higher welfare and a lower cost faces a three-way trade-off. Raising *c*_0_ raises welfare and lowers participation; it also raises the cost, unless work at some level is subsidised beyond the out-of-work income (*c_i_* − *w_i_* > *c*_0_), where the exit of subsidised workers can save more than the higher transfer costs. Raising *c_i_* raises participation and welfare, and raises the cost unless the participation tax at that level is so high that the entry it induces pays for itself. The design problems below minimise (−*P*,−*W*,*C*).

### 3.2. The Free-Schedule Problem and Its Exact Supported Pareto Front

In the free-schedule problem, FS, the planner chooses *c*_0_ ∈ [0.05, 1] and every *c_i_* ∈ [0.05, 2*w_i_* + 0.6] directly, so the decision vector has *I* + 1 components. This is the setting of optimal-tax theory with an extensive margin [20,21], and it has a property that applied problems rarely offer: the part of its Pareto front that some weighting of the three goals would choose, its supported front, can be computed exactly. For any weights *λ_P_*,*λ_W_*,*λ_C_* ≥ 0, the scalarised problem of maximising *λ_P_P* + *λ_W_W* − *λ_C_C* separates across earnings levels once *c*_0_ is fixed,

(9) maxc0[λWln c0−λCc0+∑i=1Iωimaxci{λPTai(Di)+λWΦi(Di)−λCTai(Di)(ci−wi−c0)}],

so that each point requires only nested one-dimensional searches. We solve every inner problem globally by a dense logarithmic grid followed by golden-section refinement within the bracketing grid cells, and the outer problem the same way. To check that the searches are global, we compared the solver with L-BFGS-B started from the status quo and from 20 random points on each of 40 random weightings: the best local optimum never exceeded the solver’s value by more than 5.6 × 10^−16^. Every point obtained with strictly positive weights is Pareto-optimal. A weighted sum cannot reach points on non-convex stretches of a front, so we refine the weights adaptively where neighbouring solutions lie far apart and then test the result against every point the algorithms find. The reference front consists of the 12,882 Pareto-optimal points (12,697 distinct) obtained from 12,954 weightings. None of the 26,439 final non-dominated points found by the nine algorithms in all their runs attains a higher value than the solver on any of these weightings (largest excess 1.1 × 10^−16^); 16.2% of them are not dominated by a reference point, mostly within 0.018 in normalised objective space, the 99th percentile of the nearest-neighbour spacing of the sample. The 232 points (0.9%) that lie further away fall short of the best weighted sum by no more than 0.0013 of the objective ranges (median 0.0005), so they lie on or just behind nearly flat stretches of the front that the weighted sums jump across. We also solved ε-constraint problems [43] on slices of the front: at five levels of the net cost, including that of the status quo, we maximised welfare subject to twenty lower bounds on participation and participation subject to twenty lower bounds on welfare, each problem by two independent methods that agree to within 2.5 × 10^−11^. An independent exact weighted-sum solver shows that every slice optimum meets the bound given by the weighted sums to within 5 × 10^−8^ of the objective range, so it is itself a supported point; the optima lie within 0.022 (median 0.005) of the nearest reference point, and every algorithm point lying further than 0.018 from the reference front is dominated by an ε-constraint optimum. The reference front is thus exact where it is sampled, and IGD against it measures accuracy on the exact supported front, which, on every slice and at every algorithm point checked, leaves no essential part of the Pareto front outside it. In what follows, “the exact front” is short for this exact supported front.

### 3.3. The Parametric-Instrument Problem

Real schedules are not free. In the parametric-instrument problem, PI, the planner, sets the eleven instruments of Table 2 and they generate consumption through the rules a benefit office applies. A household of type *h* with earnings *y* > 0 receives a means-tested benefit *b_h_*, pays income tax 𝒯 and may receive an in-work credit *K*.

All amounts are in units of median earnings; rates are shares.

*b_h_*(*y*) = max(0, *G* + *g_L_*𝟙[*h* = *L*] − *t_w_*max(0,*y* − *d*)),    𝒯(*y*) = *τ*max(0,*y* − *A*), (10)

*K*(*y*) = max(0, min(*r*_in_ *y*, *K*_max_) − *r*_out_max(0,*y* − *y*_po_)), (11)

and consumes

(12) $$c_{h}^{w}(y)=y-T(y)+b_{h}(y)+K(y)-\kappa (1-\sigma_{\mathrm{cc}})𝟙[h=L],{\;\;c}_{h}^{n}=G+g_{L}𝟙[h=L],$$

where *κ* is the cost of childcare that a working lone parent (*h* = *L*) must pay and *σ_cc_* the share of it the state refunds. Two household types are modelled—single adults without children (*h* = *S*) and lone parents (*h* = *L*)—each over the same *I* earnings levels and each with its own cost-of-work distribution. Participation and welfare are the population-weighted sums of Equations (6) and (7) applied to each type, with $c_{i}=c_{h}^{w}(w_{i})$ and $c_{0}=c_{h}^{n}$. In the cost Equation (8), the net transfer to a working adult is *b_h_* + *K* − 𝒯 + *κ σ_cc_*𝟙[*h* = *L*], since the part of the childcare cost that a parent pays herself is not public revenue. The maxima, the phase-out and the means test introduce kinks. Where benefit withdrawal, income tax and the phase-out of the credit together take the whole of an increase in earnings, or where a lone parent’s unrefunded childcare cost exceeds what she gains, work can fail to pay over a range of earnings. No formula characterises the optimum, and the Pareto front is not known.

### 3.4. Calibration

Table 3 lists every parameter. The behavioural parameters are chosen to be consistent with published evidence: labour-supply elasticities are relatively small, the extensive margin dominates, and among single adults the response is larger in low-income groups [17], while lone mothers respond particularly strongly [18]. We therefore set the elasticity of participation with respect to the financial gain from work, averaged over earnings levels with population weights, to 0.25 for single adults and to twice that for lone parents. These values are our choice rather than estimates, and Section 6.4 varies both. Corollary 1 then recovers the shape *n* of each type from these targets, the status quo participation profile and the status quo consumption ratios; because *n* is proportional to the target, the exponents of Table 3 are as much assumptions as the targets, and they range from 0.55 to 2.51 under the alternative targets of Section 6.4. The remaining parameters describe a stylised economy rather than a particular country; apart from the number of earnings levels, which only sets the resolution of the earnings distribution, each is varied in Section 6.4.

## 4. MOEA/D with Response-Threshold Division of Labour

MOEA/D-DoL keeps the decomposition core of MOEA/D-DE unchanged and replaces the rule that decides which subproblems are worked on in each generation by a colony of workers following the response-threshold model of Section 2. This section describes the core, the colony and the property that makes the colony allocate effort.

### 4.1. The Decomposition Core

A set of *N* uniformly spread weight vectors *λ*^1^,…,*λ^N^* defines *N* scalar subproblems through the Tchebycheff function [26]:

(13) $$g(x|\lambda^{j},z^{*})=\max_{1\leq k\leq M}\lambda_{k}^{j}|f_{k}(x)-z_{k}^{*}|,$$

where z^*^ is the best value of each objective found so far. Subproblem *j* is held by one individual **x***^j^*, and its neighbourhood *B*(*j*) contains the subproblems with the *H* nearest weight vectors. To improve subproblem *j*, MOEA/D-DE [27] draws a mating pool *Q* equal to *B*(*j*) with probability *p*_nb_ and to the whole population otherwise, and generates

(14) $$y=x^{j}+F(x^{r_{1}}-x^{r_{2}}),r_{1},r_{2}\in Q,$$

followed by polynomial mutation. The offspring replaces any member of *Q* whose Tchebycheff value it does not exceed, up to *n_r_* replacements.

### 4.2. Workers, Tasks and Thresholds

In MOEA/D-DoL individual *j* is a worker, and improving the front around subproblem *j* is its task. Unlike an ant, which may respond to several tasks, each worker here has a single task and decides only whether to work on it, which is exactly the decision of an adult in Proposition 1; the allocation of effort across subproblems emerges from these individual decisions. At the start of every generation, each worker decides independently whether to work, with the probability of Equation (1),

(15) $$\mathrm{Pr}(e_{j}(t)=1)=T_{\theta_{j}(t)}(s_{j}(t))=\frac{s_{j}(t{)}^{n}}{s_{j}(t{)}^{n}+\theta_{j}(t{)}^{n}},$$

so that it participates when its stimulus is large relative to its threshold. Every engaged worker produces one offspring by the core of Section 4.1, in random order, and its work is productive, *u_j_*(*t*) = 1, if the offspring replaces at least one member of the mating pool, that is, if it improves subproblem *j* or one of the subproblems it shares its pool with. The stimulus follows the colony rule Equation (2): the demand of a task builds up while it waits and is consumed by the work done on it,

*s_j_*(*t* + 1) = min(*s*_max_, max(0, *s_j_*(*t*) + *δ* − *α e_j_*(*t*))). (16)

The threshold follows the experiment of Ravary et al. [35], in which ants that had found prey kept on foraging while those that had always failed specialised in brood care. We read this as success lowering a worker’s threshold for its task and failure raising it. Taken literally, with fixed increments, the rule fails in an optimiser, because the rate at which offspring succeed falls by more than an order of magnitude during a run; once it drops below the break-even rate of the increments, almost every worker drifts to the maximum threshold and the division of labour dissolves (in a traced run on UF9, 90% of the workers had reached *θ*_max_ after 14,360 of 300,000 evaluations and 99% at the end, each then working in about 6% of the generations; Section 5.4 reports what this costs). We therefore measure success against the colony’s own current rate. After each generation the colony updates a smoothed success rate,

(17) $$\bar{r}(t+1)=(1-\beta )\bar{r}(t)+\beta \frac{\sum_{j}e_{j}(t)u_{j}(t)}{\sum_{j}e_{j}(t)},$$

and then each worker’s threshold, which moves only when the worker works,

(18) $$\theta_{j}(t+1)=\mathrm{clip}(\theta_{j}(t)-\xi e_{j}(t)u_{j}(t)+\xi \frac{\hat{r}}{1-\hat{r}}e_{j}(t)(1-u_{j}(t)),\theta_{\min},\theta_{\max}),\;\;\;\hat{r}=\mathrm{clip}(\bar{r}(t+1),10^{-4},0.95),$$

idle workers keeping theirs, as the division of labour in the experiment was a lasting one. The clip on $\hat{r}$ only keeps the failure increment finite.

**Lemma** **1.** *If a working worker succeeds with probability r_j_ and the colony rate is* $\hat{r}$*, the expected change of its threshold is*

(19) $$E[\theta_{j}(t+1)-\theta_{j}(t)|e_{j}(t)=1]=\xi \frac{\hat{r}-r_{j}}{1-\hat{r}},$$

*ignoring the clip on θ_j_ and the influence of the worker’s own outcome on *$\hat{r}$*, which is at most *$\beta /\sum_{k}e_{k}(t)$.

**Proof.**  The expectation is $-\xi r_{j}+\xi \;\hat{r}(1-r_{j})/(1-\hat{r})=\xi \;[\hat{r}(1-r_{j})-r_{j}(1-\hat{r})]/(1-\hat{r})=\xi (\hat{r}-r_{j})/(1-\hat{r})$. □

Workers that succeed more often than the colony therefore become specialists whose thresholds fall towards *θ*_min_ and who work whenever their stimulus has recovered; workers that succeed less often become reluctant, and work only when their task has waited long enough for its stimulus to saturate. Which way a threshold drifts depends only on how a worker compares with its colony, not on how high the colony’s success rate is, so the sorting of workers continues as success becomes rarer, although, by Equation (19), more slowly. The stimulus supplies the other half of the behaviour. A worker whose stimulus is used up cannot work, since *T_θ_*(0) = 0, so work is rationed by the rate *δ* at which the stimulus is replenished: with the values used below, a specialist at *θ*_min_ works in half of the generations, and a reluctant worker at *θ*_max_ still works in about one in seventeen, so no task is monopolised and none is abandoned. Section 5.3 shows the division of labour that results.

**Figure 3:**
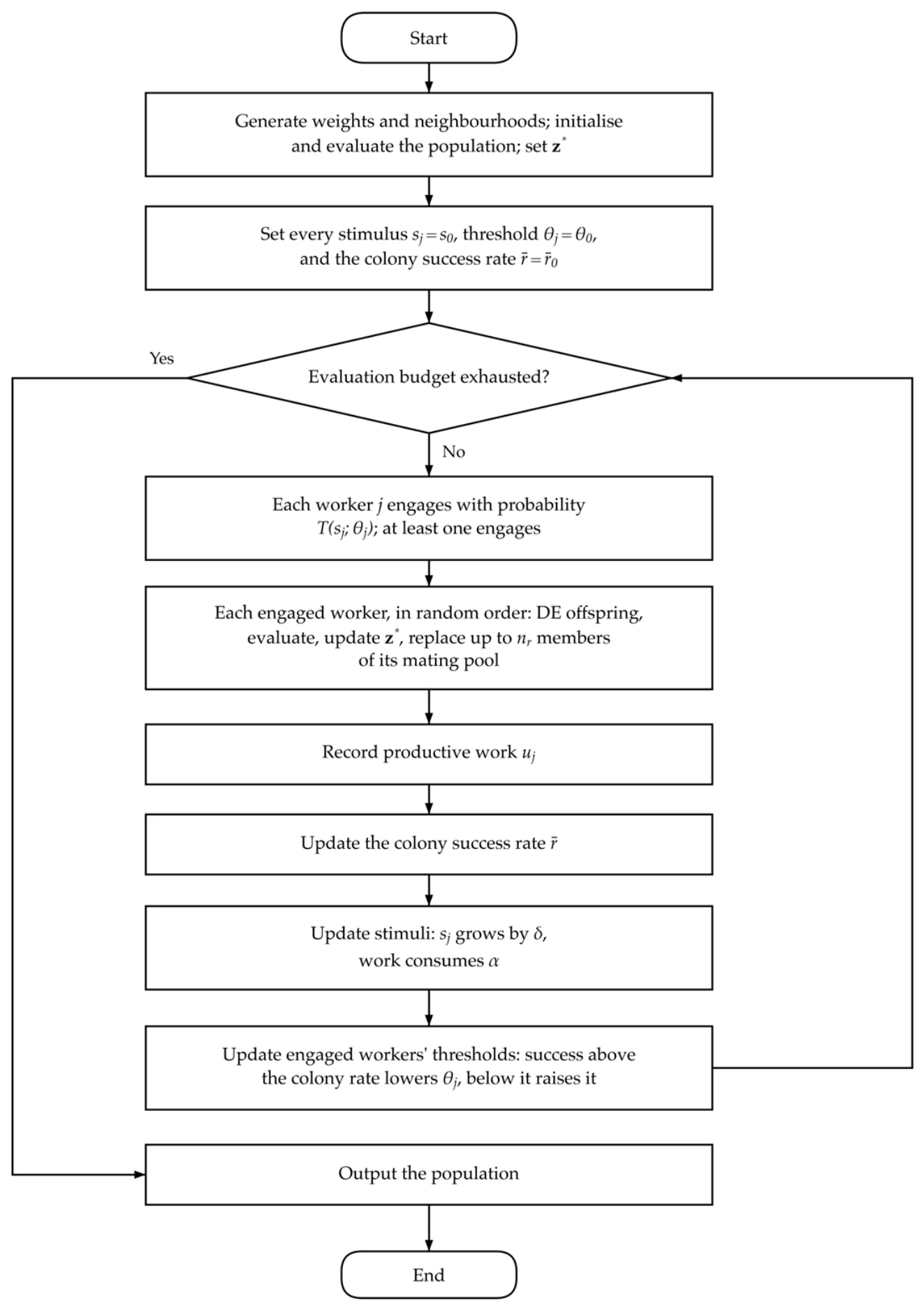
Flowchart of MOEA/D-DoL; z* is the reference point of Equation (13), the best value of each objective found so far.

Algorithm 1 gives the procedure and Figure 3 its flow. Some values were never compared in a pilot: the exponent *n* = 2, which sets only the steepness of the response (a symmetric sigmoid in ln *s* for every *n*) and was kept from the first pilot; the ranges *s* ∈ [0, 1] and *θ* ∈ [0.05, 4], *θ*_max_ having been raised from 1 to 4 when the threshold rule was changed (Appendix B); the initial values *s_j_*(0) = *θ_j_*(0) = 1, at which every worker starts with an even chance of working; and *β* = 0.1 and *r*(0) = 0.5 for the colony’s smoothed success rate. The rates *δ*, *α* and *ξ* were chosen in pilot runs with seeds that the reported runs do not use (101–110) on eleven of the benchmark problems (UF1–UF4, UF7–UF9, DTLZ1, DTLZ3, DTLZ4 and DTLZ7). In the last pilot, on nine of them, four settings of *δ* and *ξ* at *α* = 0.2 were compared, and we kept *δ* = 0.1 and *ξ* = 0.1, which shared the best median-IGD rank and, unlike the other setting with that rank, had no significant loss against MOEA/D-DE; the other three settings had a 2–6% lower geometric-mean median IGD, so the choice rests on this selection rule rather than on accuracy. Table A4 in Appendix B documents the pilots, and Section 5.5 varies every rate, the ratio *δ*/*α* and the exponent *n*.
**Algorithm 1:** MOEA/D with Response-Threshold Division of Labour (MOEA/D-DoL)1: *Input:* problem, population size *N*, evaluation budget, neighbourhood size *H*.2: Generate weights *λ*^1…*N*^ and neighbourhoods *B*(*j*); initialise **x**^1…*N*^ at random, evaluate, set z^*^.3: Set *s_j_* ← *s*_0_, *θ_j_* ← *θ*_0_$\;\mathrm{for}\;\mathrm{every}\;\mathrm{worker}\;j,\;\mathrm{and}\;\bar{r}\leftarrow {\bar{r}}_{0}$.4: *while* the budget is not exhausted *do*5:  $\mathrm{Draw}\;e_{j}∼\mathrm{Bernoulli}(T_{\theta_{j}}(s_{j}))$ for every *j* by Equation (15); if no *e_j_* = 1, set one at random.6:  *for* each engaged worker *j*, in random order, *do*7:   Draw the mating pool *Q*; generate **y** by Equation (14) and polynomial mutation; evaluate; update z^*^.8:   Replace up to *n_r_* members of *Q* that **y** does not worsen; set *u_j_* ← 1 if any was replaced, else *u_j_* ← 0.9:  *end for*10:   $\mathrm{Update}\;\bar{r}$ by Equation (17), every stimulus by Equation (16) and every threshold by Equation (18).11:  *end while*12:  *Output:* the population **x**^1…*N*^.

### 4.3. Cost

The colony adds a handful of vector operations of length *N* per generation to MOEA/D-DE’s expected cost of *O*(*M*[*p*_nb_*H* + (1 − *p*_nb_)*N*]) per offspring, and it spends exactly the same number of evaluations: a generation in which fewer workers engage is simply cheaper, so more generations fit into the budget. Measured over the benchmark runs, the median wall-clock time of MOEA/D-DoL was 1.01 times that of MOEA/D-DE (geometric mean over the seventeen problems, range 0.90–1.06).

## 5. Evaluation on Benchmark Problems

Before MOEA/D-DoL is used on the policy problems it has to earn its place on problems whose answers are known. This section compares it with eight established algorithms, takes the colony apart to see which of its parts matter, and watches the division of labour form during a run.

### 5.1. Protocol

The main test set has seventeen problems. The ten unconstrained problems of the CEC 2009 competition, UF1–UF10 [44], have 30 decision variables and complicated Pareto sets, two objectives in UF1–UF7 and three in UF8–UF10. The seven DTLZ problems [45] are used with three objectives and 7, 12 and 22 variables for DTLZ1, DTLZ2–DTLZ6 and DTLZ7; they add multimodality (DTLZ1, DTLZ3), a biased density of solutions (DTLZ4), degenerate fronts (DTLZ5, DTLZ6) and a disconnected front (DTLZ7). As a held-out suite, none of whose problems was used in designing MOEA/D-DoL, we add the nine WFG problems [46] with three objectives, four position-related and twenty distance-related variables and a budget of 100,000 evaluations; they add non-separable, deceptive and biased problems and mixed front shapes. Their reference fronts are sampled as in PlatEMO (5050 points; for WFG2, whose dominated points are removed, the lattice is enlarged to 7260 points so that 5466 non-dominated points remain), except that of WFG3: with three objectives, its front is not the degenerate line that PlatEMO samples [47], so it is represented by 4667 points of the true front. Accuracy is measured by the inverted generational distance(IGD), the mean distance from a dense sample of the true front to the nearest obtained point [44], computed on the non-dominated subset of the final population. The hypervolume [48], computed after the ideal and nadir points of the true front are mapped to 0 and 1 and with the reference point at 1.1 in every objective, is reported as a second view. The samples of the true fronts of these seventeen problems have between 21 points (UF5, whose front is 21 isolated points) and 5050 points.

Table 4 lists the comparison algorithms. They cover three families: Pareto-dominance and reference-point algorithms (NSGA-II [49], NSGA-III [50], RVEA [51] and AR-MOEA [52]), decomposition algorithms (MOEA/D-DE [27] and MOEA/D-FRRMAB [30]) and swarm algorithms (MOPSO [53] and the multi-objective grey wolf optimiser, MOGWO [54]). MOEA/D-DE is the core on which MOEA/D-DoL is built. The two differ in the colony and in the order in which subproblems are served, fixed in MOEA/D-DE and random for the engaged workers of MOEA/D-DoL; an ablation in Section 5.4 separates the two. MOEA/D-FRRMAB chooses among four differential-evolution operators with a multi-armed bandit; its PlatEMO implementation, which we follow, also picks the subproblems to work on by a utility of their recent improvement, which makes it the comparator closest to an engineered answer to the allocation question.

All algorithms were implemented in Python (version 3.13) for this study. The problem definitions and the eight comparison algorithms were checked line by line against the source code of the MATLAB platform PlatEMO (https://github.com/BIMK/PlatEMO, accessed on 21 October 2026), except MOGWO, which PlatEMO does not include and which follows its original publication. Every algorithm has a population of 100 on two-objective and 105 on three-objective problems, and a budget of 300,000 evaluations on UF1–UF10, the budget of the CEC 2009 competition, and 100,000 on DTLZ1–DTLZ7. Each algorithm was run 30 times on every problem with seeds 1–30, and for a given seed, all algorithms started from the same initial population; the pilot runs that fixed the design of MOEA/D-DoL used seeds 101–110 and are reported only in Appendix B. The evaluation budget is the only stopping rule: the decomposition algorithms stop exactly at it and the generational algorithms when the next generation would exceed it, at most 0.055% short. The comparison algorithms keep the settings of Table 4, without tuning on these problems, whereas the colony parameters of MOEA/D-DoL were chosen in pilot runs (Section 4.2), an advantage on the pilot problems that Section 5.2 takes into account. For each problem and indicator, the 30 final values of MOEA/D-DoL are compared with the 30 values of each comparison algorithm by a two-sided Wilcoxon rank-sum test; the eight *p*-values of a problem form one family adjusted by Holm’s procedure, and the four ablation variants of Section 5.4 form a separate family. In the tables, + (−) marks an algorithm significantly worse (better) than MOEA/D-DoL at an adjusted level of 0.05, the direction being given by the medians, and ≈ no significant difference. All summaries are medians with interquartile ranges, and the algorithms are ranked over all problems by the Friedman test on median IGD with Nemenyi post hoc comparisons [55]. Because a rank-sum test declares small but consistent differences significant, we call a significant difference negligible when the two medians differ by less than 1%. The rankings of metaheuristics can depend on the evaluation protocol [39], and reproducible comparisons fix the budget, the number of runs and the indicators in advance, and report several complementary measures [56]; the budget, the number of runs and the indicators were fixed before the reported runs and apply unchanged to the WFG suite and the policy problems, whereas the 1% threshold for negligible differences and the ranking by median IGD were adopted after the runs had been made.

### 5.2. Accuracy

**Figure 4:**
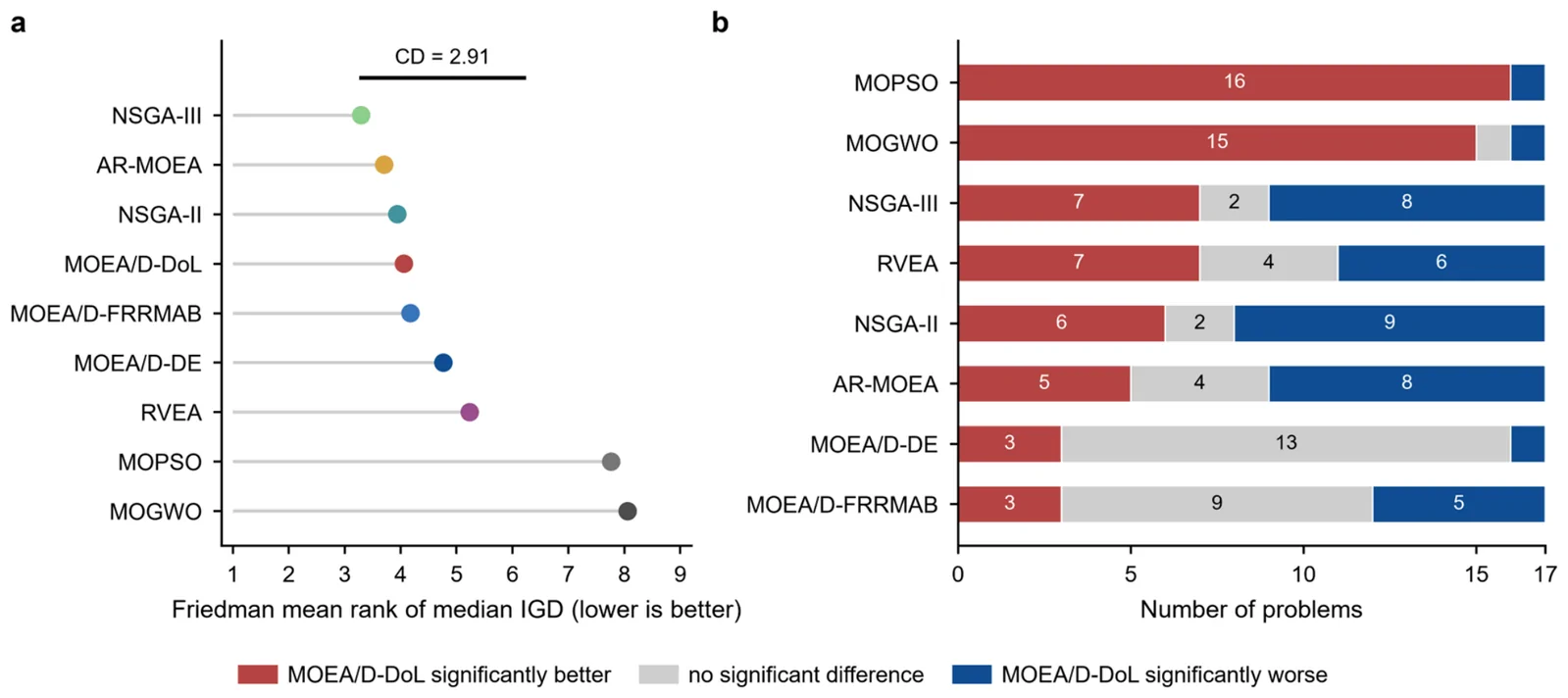
Overall comparison on the benchmark problems: (**a**) Friedman mean ranks by median IGD; (**b**) Wilcoxon outcomes of MOEA/D-DoL against each algorithm.

Figure 4 summarises the comparison on the seventeen problems, and Table A1 and Table A2 in Appendix A give the full statistics. By the Friedman test on median IGD, the nine algorithms differ (*χ*^2^ = 55.3, *p* = 4 × 10^−9^). NSGA-III has the best mean rank (3.29), followed by AR-MOEA (3.71) and NSGA-II (3.94); MOEA/D-DoL ranks fourth (4.06), ahead of MOEA/D-FRRMAB (4.18) and MOEA/D-DE (4.76), and only MOPSO and MOGWO are separated from NSGA-III by more than the Nemenyi critical difference of 2.91. Against its own core, MOEA/D-DoL is significantly better than MOEA/D-DE on three problems (UF2, UF7 and DTLZ6), not significantly different on thirteen and worse on one (UF3). The gains on UF2 and UF7 lower the median IGD by 16% and 29%, whereas that on DTLZ6 is negligible (0.055%); by hypervolume MOEA/D-DoL is better on UF2, UF7, DTLZ5 and DTLZ6 and worse on none, all four differences being below 1%. Against MOEA/D-FRRMAB it is better on three problems by IGD, equal on nine and worse on five. Eleven of the seventeen problems were used in the pilot runs that shaped MOEA/D-DoL (Section 4.2); both of its non-negligible gains are among them, and on the six held-out problems (UF5, UF6, UF10, DTLZ2, DTLZ5 and DTLZ6) its only significant differences from MOEA/D-DE are negligible ones (IGD on DTLZ6, hypervolume on DTLZ5 and DTLZ6).

**Figure 5:**
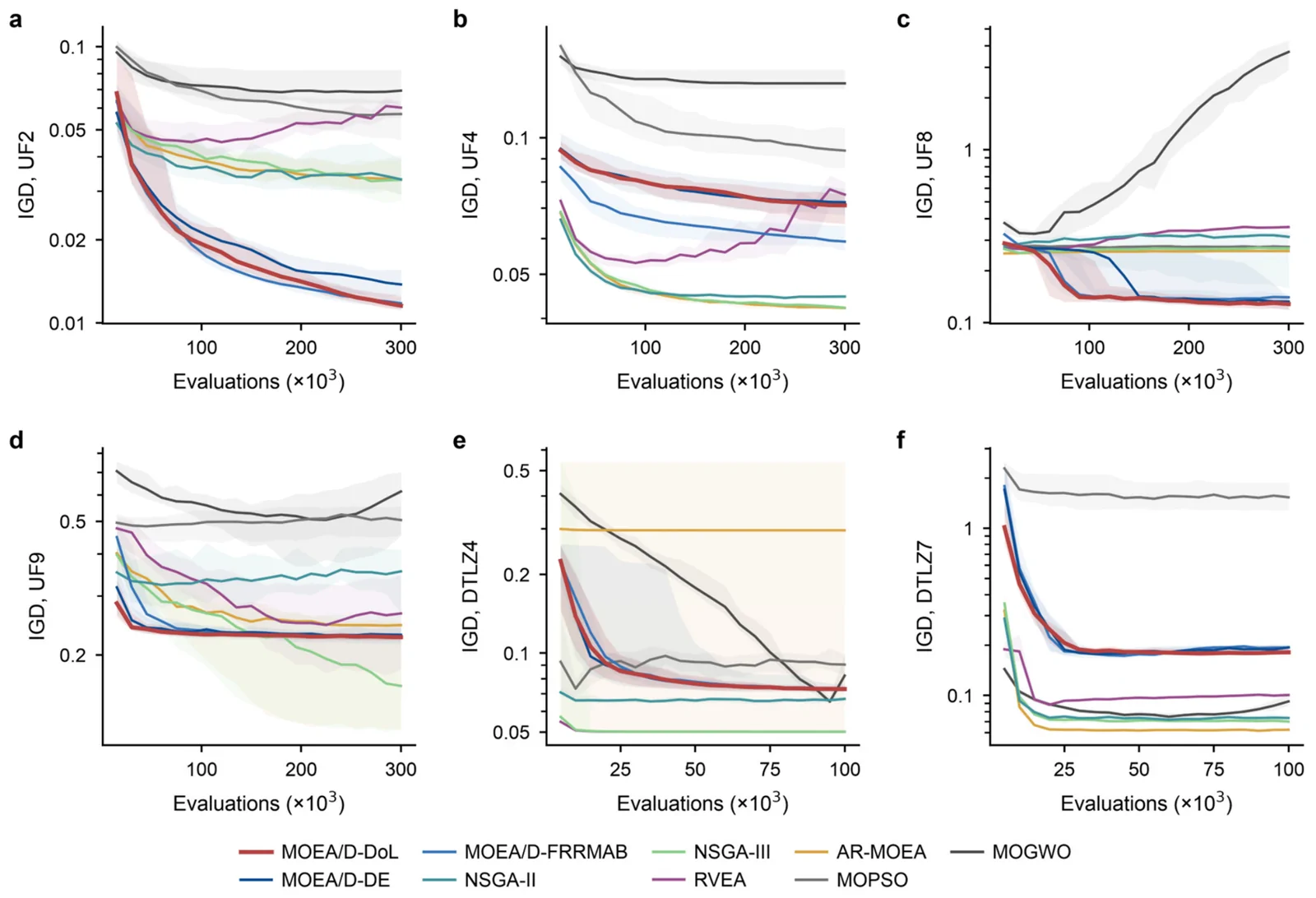
Median IGD against evaluations on six benchmark problems: (**a**) UF2; (**b**) UF4; (**c**) UF8; (**d**) UF9; (**e**) DTLZ4; (**f**) DTLZ7. Shaded bands show the interquartile range over 30 runs.

Means and medians can disagree because of occasional failed runs, whose IGD is more than twice the median of the two algorithms’ pooled runs; this is why all tables report medians. Over the 17 problems, MOEA/D-DoL has 30 such runs and MOEA/D-DE 28, but these runs are distributed differently over the problems: MOEA/D-DoL fails more often on UF2 (4 against 0), UF3 (8 against 3), UF6 (3 against 1) and UF7 (7 against 5); less often on UF8 (4 against 6), DTLZ3 (0 against 1), DTLZ4 (1 against 6) and DTLZ7 (2 against 5); and equally often on UF1 (1 each). On UF2, for instance, four failed runs put the mean IGD of MOEA/D-DoL above MOEA/D-DE’s, although its median is 16% lower. Against NSGA-II, NSGA-III and AR-MOEA, the balance is negative: MOEA/D-DoL is better on 6, 7 and 5 problems and worse on 9, 8 and 8; against RVEA, it is better on 7 and worse on 6, and it is better than MOPSO and MOGWO on 16 and 15 of the 17. Figure 5 shows how the median IGD develops on six problems that span the suite.

To see whether the colony speeds up convergence without choosing a problem or a budget after looking at the curves, we compared MOEA/D-DoL with MOEA/D-DE at 25%, 50% and 75% of the budget on all seventeen problems, with Holm’s correction over the problems at each checkpoint. Only two differences are significant: on UF1 at a quarter of the budget (median IGD 0.017 against 0.022) and, by a negligible margin, on DTLZ6 at three-quarters. An apparent advantage on UF8 at a quarter of the budget, visible in Figure 5 (0.164 against 0.265, unadjusted *p* = 0.004), does not survive the correction (adjusted *p* = 0.06). At the full budget, with the same correction over problems, UF7 and DTLZ6 remain significant but UF2 does not (adjusted *p* = 0.055). On UF2 and UF7 the advantage builds late: the median IGD of MOEA/D-DoL falls to MOEA/D-DE’s final median only between 65% and 70%, and between 55% and 60%, of the budget.

### 5.3. The Division of Labour During a Run

**Figure 6:**
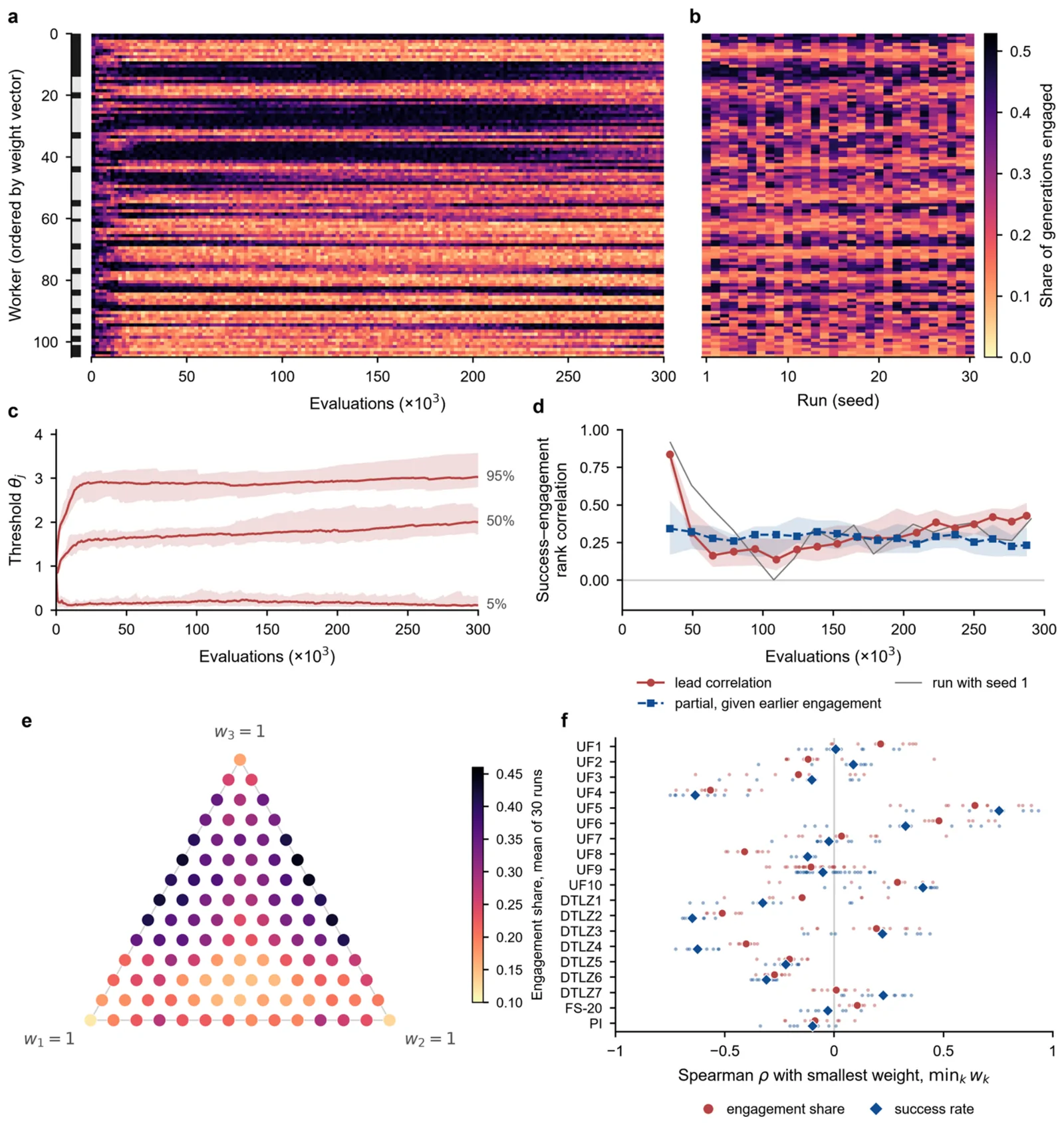
Division of labour in MOEA/D-DoL: (**a**,**b**) UF9 engagement; (**c**) thresholds; (**d**) success–engagement correlation; (**e**) engagement by weight; (**f**) edge correlation. Shaded areas show the range across the 30 UF9 runs in (**c**) and the interquartile range across runs in (**d**); in (**f**), small dots are single runs and large markers medians over runs.

Figure 6 follows the division of labour of MOEA/D-DoL on UF9, a three-objective problem with a complicated Pareto set, in all 30 runs. In most runs the colony splits early. Across workers, the busiest 5% engaged in 49% of the generations (median over runs; 48–50%), close to the most a worker can under the stimulus rule, and the least busy 5% in 10% (9–12%); the spread of thresholds reached 90% of its later level after a median of 11,300 evaluations, within 30,000 evaluations in 25 of the runs and after 34,000 to 141,000 in the other five, with final 5%, 50% and 95% quantiles of 0.11, 2.01 and 3.03 (Figure 6c). Figure 6a shows the engagement of each worker in one run and Figure 6b its engagement over the whole of each run: some bands recur from run to run (rank correlation of the workers’ engagement between runs 0.46; intraclass correlation 0.44), so part of the division of labour is tied to the subproblem rather than to the run.

What sorts the workers is how their success compares with the colony’s, as Lemma 1 predicts. Over windows of 500 generations, the rank correlation between a worker’s success relative to the colony in one window and its engagement in the next was significantly positive in 437 of the 578 windows of the 30 runs and significantly negative in none (Figure 6d); the partial correlation, with the worker’s engagement in the earlier window held fixed, is positive in 568 of the 578 windows and significantly positive in 419, so it is not merely persistence. Workers whose success over 1000 generations was above the colony’s raised their engagement in the next 1000 by 1.0 percentage point (median over runs), while those below it lowered it by 2.1 points and those without any success by 1.7. The colony’s success rate meanwhile fell by a factor of 19 (median over runs), from 0.16 in the first thousand generations to 0.0083 in the last. Over all 210 traced runs (30 on UF9 and 10 on each of the other sixteen benchmarks and the two policy problems), the mean lead correlation is positive in every run, and 3472 of 4050 windows are significantly positive and none negative.

Does the colony simply send its effort to easy subproblems at the edges of the front? Figure 6e shows the mean engagement of each worker over the 30 runs, placed at its weight vector. Effort is only slightly tilted towards the edges: the rank correlation between a worker’s engagement and its smallest weight component is −0.11 (median over runs; negative in 24 of 30), edge workers do 1.06 times their proportional share of the work, and success rates show no edge effect (−0.05); the stable pattern is one of direction, with workers that weight the third objective heavily busier than the rest. Over all 19 traced problems, the sign of this correlation varies (negative on 11, positive on 8; Figure 6f), so the colony has no general preference for the edges. The edge-heavy coverage of the decomposition algorithms on the free-schedule problem (Section 6.1) therefore does not come from the colony: MOEA/D-DE, which serves every subproblem equally, places 89% of its points within a normalised distance of 0.05 of the boundary of that front, exactly as MOEA/D-DoL does.

### 5.4. Ablation

Table 5 removes one part of the colony at a time. DoL-fixed keeps the stimulus but freezes every threshold, which is the fixed-threshold model of Section 2.1; DoL-abs replaces the comparison with the colony by the literal reinforcement rule; DoL-random keeps the average amount of work but chooses the workers blindly, each engaging with the constant probability *q* = 0.21, the mean engagement rate of MOEA/D-DoL over the nine problems of the last pilot; and DoL-all engages every worker in every generation in random order, which differs from MOEA/D-DE only in the order in which subproblems are served.

Each ablation is significantly worse than the full algorithm on two or three problems, one of which, DTLZ6, by a negligible margin (medians within 0.1%), and not significantly different on the other fourteen or fifteen, so on most problems, the parts of the colony make no detectable difference. Where the full algorithm gains over MOEA/D-DE, on UF2, UF7 and DTLZ6, no variant keeps a significant gain, and every variant except DoL-fixed is significantly worse than the full algorithm on all three; DoL-fixed is worse on UF7 and DTLZ6 but not on UF2 (adjusted *p* = 0.08), so the gain on UF2 cannot be attributed to threshold learning. Over all seventeen problems, a Wilcoxon signed-rank test on the problem medians finds the full algorithm better than DoL-abs and DoL-random (Holm-adjusted *p* = 0.033 and 0.022) but not than DoL-fixed (*p* = 0.17) or DoL-all (*p* = 0.07); by the same test, without adjustment, the full algorithm is not significantly better than MOEA/D-DE over the seventeen problems (*p* = 0.15). Serving the subproblems in random order without a colony (DoL-all) is significantly better than MOEA/D-DE on none of the seventeen problems, so the visiting order does not explain the gains. As a geometric mean of median IGD relative to MOEA/D-DE, the full algorithm stands at 0.987, the variant with fixed thresholds at 0.997, and the variants that drop the comparison with the colony, the choice of workers or the division of labour at 1.040, 1.047 and 1.041. Two of the three problems on which the colony gains, UF2 and UF7, were used in its design (Section 5.2), and the gain on the third is negligible.

### 5.5. Parameter Sensitivity

**Figure 7:**
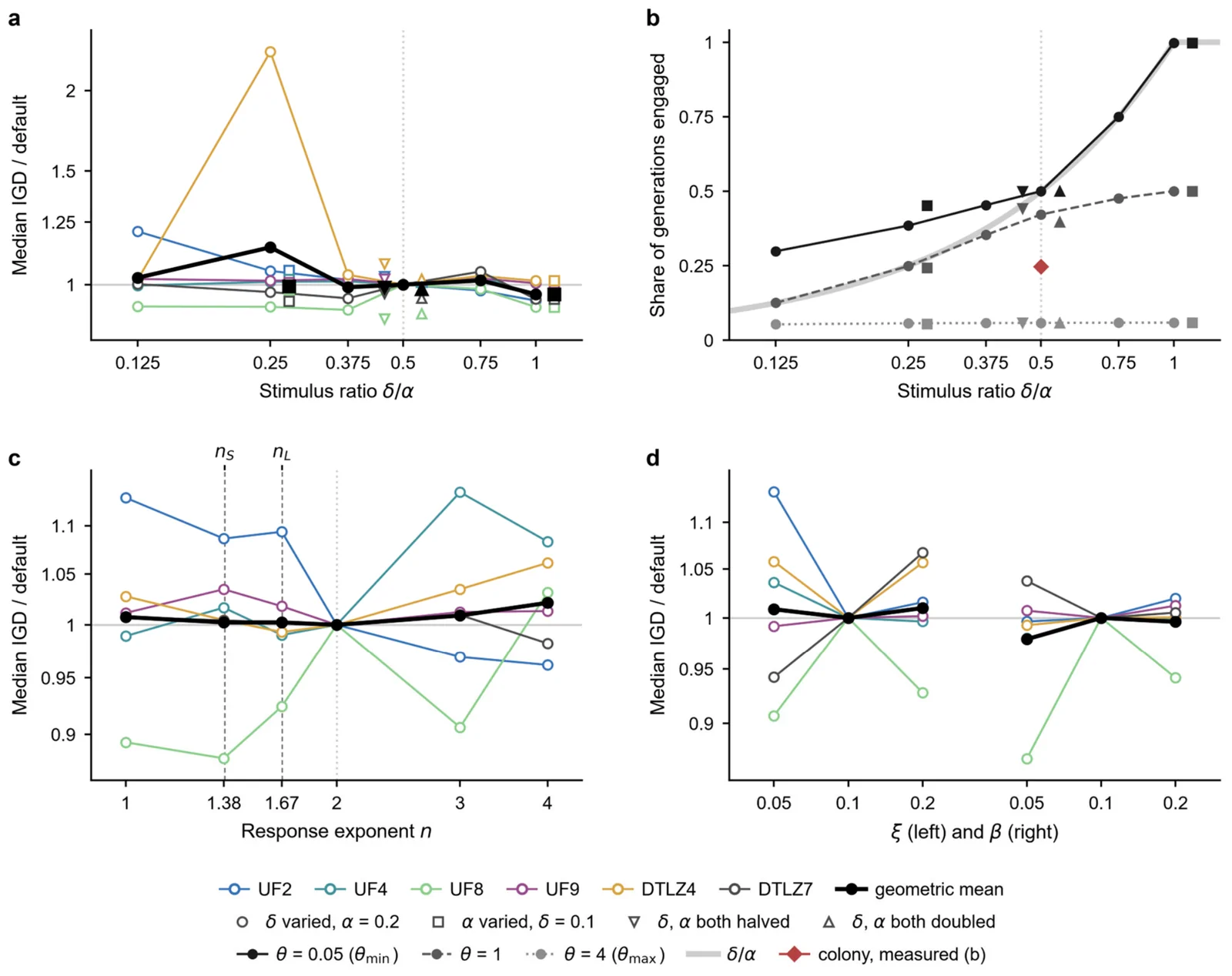
Sensitivity of MOEA/D-DoL on six problems: (**a**) relative median IGD and (**b**) engagement against *δ*/*α*; (**c**) exponent *n*; (**d**) *ξ* and *β*. Open coloured symbols are single problems and filled black symbols their geometric mean; symbol shapes show how *δ*/*α* is reached; in (**b**), filled black and grey symbols give the engagement of a worker with *θ* = 0.05, 1 and 4, and the red diamond the engagement measured in the colony.

Figure 7 varies the colony parameters on six problems with ten runs each (seeds 1–10) and reports the median IGD relative to the default setting on the same seeds; all these runs, the default included, were repeated on one computer for this section. Because a worker’s stimulus grows by *δ* in every generation and falls by *α* when it works, the ratio *δ*/*α* governs how often a worker can work: a worker that has to wait for its stimulus to build up works in a share of generations close to *δ*/*α*, while a specialist, whose stimulus is only cut to zero, can work more often than that when the ratio is small (Figure 7b). *δ* and *α* are therefore varied along this ratio, from 0.125 to 1 (the default is 0.5), and at the default ratio with both halved or both doubled; *ξ* and *β* are set to half and twice their values, and the response exponent *n* to 1, 1.38, 1.67, 3 and 4, where 1.38 and 1.67 are the labour-market exponents of Table 3.

No setting changed the median IGD significantly on any of the six problems (rank-sum tests against the default, Holm-corrected over the eighteen settings). Averaged over the problems as a geometric mean, the ratios lie between 0.97 and 1.02, except at *δ*/*α* = 0.25 reached by halving *δ* (1.14). That exception comes from DTLZ4, where the median IGD rises 2.3-fold because five of the ten runs, against one with the default, cover only part of the front, the known difficulty of this biased problem; reaching the same ratio by doubling *α* leaves DTLZ4 unchanged (0.997, three such runs), and neither difference is significant. The ratio acts through engagement (Figure 7b): a worker with threshold 1 works in a share of the generations close to *δ*/*α* up to 0.375 (0.42 at the default), a specialist at *θ*_min_ in 30% to 100% of them as the ratio rises from 0.125 to 1, and a reluctant worker at *θ*_max_ in about one in seventeen throughout. At *δ*/*α* = 1, the stimulus never falls below its maximum, and engagement depends on the thresholds alone, so doubling *δ* and halving *α* give identical runs, as they must. The exponent *n* mainly sets how strongly reluctant workers are held back, from one generation in five at *n* = 1 to about one in 260 at *n* = 4, yet the geometric-mean ratios stay between 1.00 and 1.02, and at 1.002 for both labour-market exponents (Figure 7c). Within these ranges the results do not depend on a finely tuned setting.

### 5.6. Held-Out Test Problems

Table A3 in Appendix A reports the nine held-out WFG problems, scored as the other benchmarks with 30 runs per algorithm. Against its core, MOEA/D-DoL is significantly better on WFG1 and WFG4, where it lowers the median IGD by 9.5% and 1.1%, and by a negligible margin (0.2%) on WFG5; it is not significantly different on the other six problems and worse on none, and neither algorithm has a failed run. By hypervolume it is better on six problems, by more than 1% on WFG1, WFG4 and WFG8, and worse on none. The gains of more than 1% (IGD on WFG1 and WFG4, hypervolume on WFG1, WFG4 and WFG8) are the first non-negligible gains of the colony on problems that played no part in its design; on the six held-out problems of the main set, its gains were negligible (Section 5.2). They do not change its standing among the algorithms, however. By the Friedman test on median IGD over the nine problems (*χ*^2^ = 42.8, *p* = 1.0 × 10^−6^), MOEA/D-DoL ranks fifth (5.33), ahead of MOEA/D-FRRMAB (5.78) and MOEA/D-DE (5.89) but behind AR-MOEA (2.33), NSGA-III (2.89), RVEA (3.11) and NSGA-II (3.78); with nine problems, the Nemenyi critical difference of 4.00 separates none of them from it. NSGA-II and NSGA-III are significantly more accurate than MOEA/D-DoL on seven of the nine problems, and AR-MOEA on eight.

## 6. Designing Participation Policy

The two problems of Section 3 were solved by all nine algorithms and the ablation variants, 30 runs each with seeds 1–30, a population of 105 and 100,000 evaluations. The normalisation diagnostic of Section 6.1 (ten runs per algorithm), the marginal-rate cap and wider ranges of Section 6.3 (30 AR-MOEA runs each) and the calibration study of Section 6.4 (ten AR-MOEA runs per calibration) lie outside this comparison. Because the three objectives are measured in different units, IGD and hypervolume are computed after each objective is scaled to [0, 1] by the ideal and nadir points of the reference front. For FS the reference front is the exact one of Section 3.2; for PI, whose front is unknown, it is the non-dominated union of the final populations of all runs of all algorithms, so that PI scores measure how close each algorithm comes to the best that any of them found.

### 6.1. Accuracy Against the Exact Supported Front

Table 6 reports the accuracy of every algorithm on the two problems. On FS, measured against the exact front, AR-MOEA (median 0.052), NSGA-II (0.061), NSGA-III (0.067) and RVEA (0.105) are more accurate than the decomposition algorithms. The three decomposition algorithms and the four ablation variants lie together between 0.135 and 0.138, and MOEA/D-DoL differs significantly from none of them; MOPSO (0.194) and MOGWO (0.264) trail. Each of the more accurate algorithms is significantly better than MOEA/D-DoL. By hypervolume, which rewards the extremes of a front, the order is different: NSGA-II (0.898) and AR-MOEA (0.894) come first, followed by the decomposition algorithms and ablation variants (0.881–0.883), ahead of NSGA-III (0.856), RVEA (0.855), MOPSO (0.613) and MOGWO (0.597).

**Figure 8:**
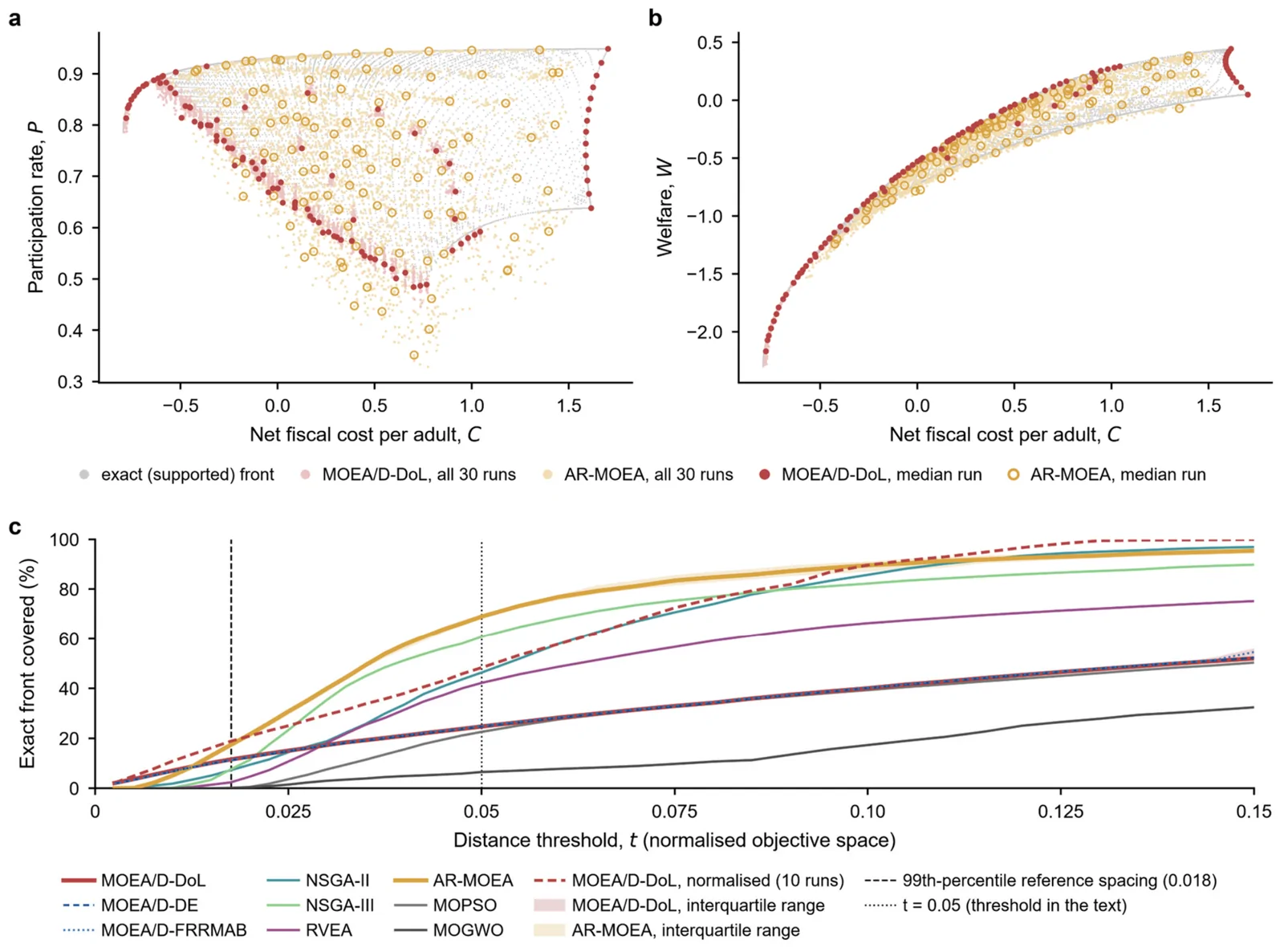
Exact supported free-schedule front with MOEA/D-DoL and AR-MOEA points: (**a**) participation and (**b**) welfare against cost; (**c**) coverage against distance.

The gap is one of coverage rather than convergence. Figure 8 plots the final points of all 30 MOEA/D-DoL and AR-MOEA runs, with the median run of each highlighted, on top of the exact front, and the coverage of the front by every algorithm (and by the normalised MOEA/D-DoL) as a function of the distance *t*. The decomposition algorithm places its points along the edges of the front, where one objective is at an extreme, and leaves most of the interior empty; AR-MOEA spreads its points over much of the interior but misses the low-cost end, which the decomposition algorithm covers, and some of its points are still short of the front. Measured as the share of the exact front that has a found point within a normalised distance *t*, the three decomposition algorithms cover 25% of it at *t* = 0.05 (median over runs), against 69% for AR-MOEA, 61% for NSGA-III and 46% for NSGA-II (Figure 8c). At short distances the order reverses for every Pareto-based and reference-point algorithm: at *t* = 0.01 the decomposition algorithms cover more (7%, against 5% for AR-MOEA and 2% or less for NSGA-II, NSGA-III and RVEA), because their points lie closest to the front. AR-MOEA covers more than any decomposition algorithm from *t* = 0.015 (18% against 11–12% at *t* = 0.018, the 99th percentile of the spacing of the reference sample), NSGA-III from *t* = 0.0225, NSGA-II from *t* = 0.0275 and RVEA from *t* = 0.03, and at *t* = 0.10, AR-MOEA covers 90% against 40%. The points they do find lie closer to the front: their mean normalised distance to it is 0.0076–0.0100 (means over runs), against 0.0288 for AR-MOEA, 0.0376 for NSGA-III and 0.0495 for NSGA-II, and MOEA/D-DoL’s (0.0076) is significantly smaller than MOEA/D-DE’s (0.0096; rank-sum *p* = 0.0003).

The scaling of the objectives accounts for most of the gap. The Tchebycheff function Equation (13) compares objectives in their own units, and on the exact front of FS, the ranges of participation, welfare and cost are 0.46, 2.75 and 2.50, so uniformly spread weight vectors are far from uniformly spread over the front; AR-MOEA and NSGA-III normalise the objectives before they compare solutions. Normalising the objectives inside the decomposition is not new: the original MOEA/D paper showed that it makes the solutions far more uniform when the objectives are disparately scaled [26]. As a diagnostic outside the comparison, we reran MOEA/D-DE and MOEA/D-DoL with seeds 1–10 and each objective divided by its current range in the population inside Equation (13). On FS the median normalised IGD fell from 0.138 to 0.054 for MOEA/D-DE and from 0.137 to 0.054 for MOEA/D-DoL (rank-sum *p* = 1.8 × 10^−4^ for each, normal approximation; exact *p* = 1.1 × 10^−5^), and the covered share of the front rose from 25% to 48% at *t* = 0.05, and to about 90% at *t* = 0.10. AR-MOEA, at 0.052 and 69% on the same seeds, remains significantly more accurate than both (rank-sum *p* = 0.007 and 0.014). On PI the normalised runs reach 0.0362 (MOEA/D-DE) and 0.0364 (MOEA/D-DoL), against 0.0370 for AR-MOEA on the same seeds (rank-sum *p* = 0.017 and 0.054). With normalisation, MOEA/D-DoL and MOEA/D-DE show no significant difference (*p* = 0.62 on FS and 0.21 on PI, ten runs each). On these problems, the decomposition, not the allocation of effort among its subproblems, limits accuracy.

These differences matter to a planner. At the status quo cost of the free schedule, the highest participation rate in the final population of a single run is 86.3% for AR-MOEA (median of the 27 runs that have a schedule at that cost) and 67.7% for MOEA/D-DoL (median of 30 runs), against 93.2% on the exact front and 73.5% at the status quo; the decomposition algorithms find, at that cost, only schedules that trade participation for welfare. A planner relying on one run of a decomposition algorithm would therefore miss the participation gains that are available at no extra cost, whereas the choice between MOEA/D-DoL and its core, which differ there by 0.4 percentage points, would not matter.

### 6.2. Results on the Parametric-Instrument Problem

**Figure 9:**
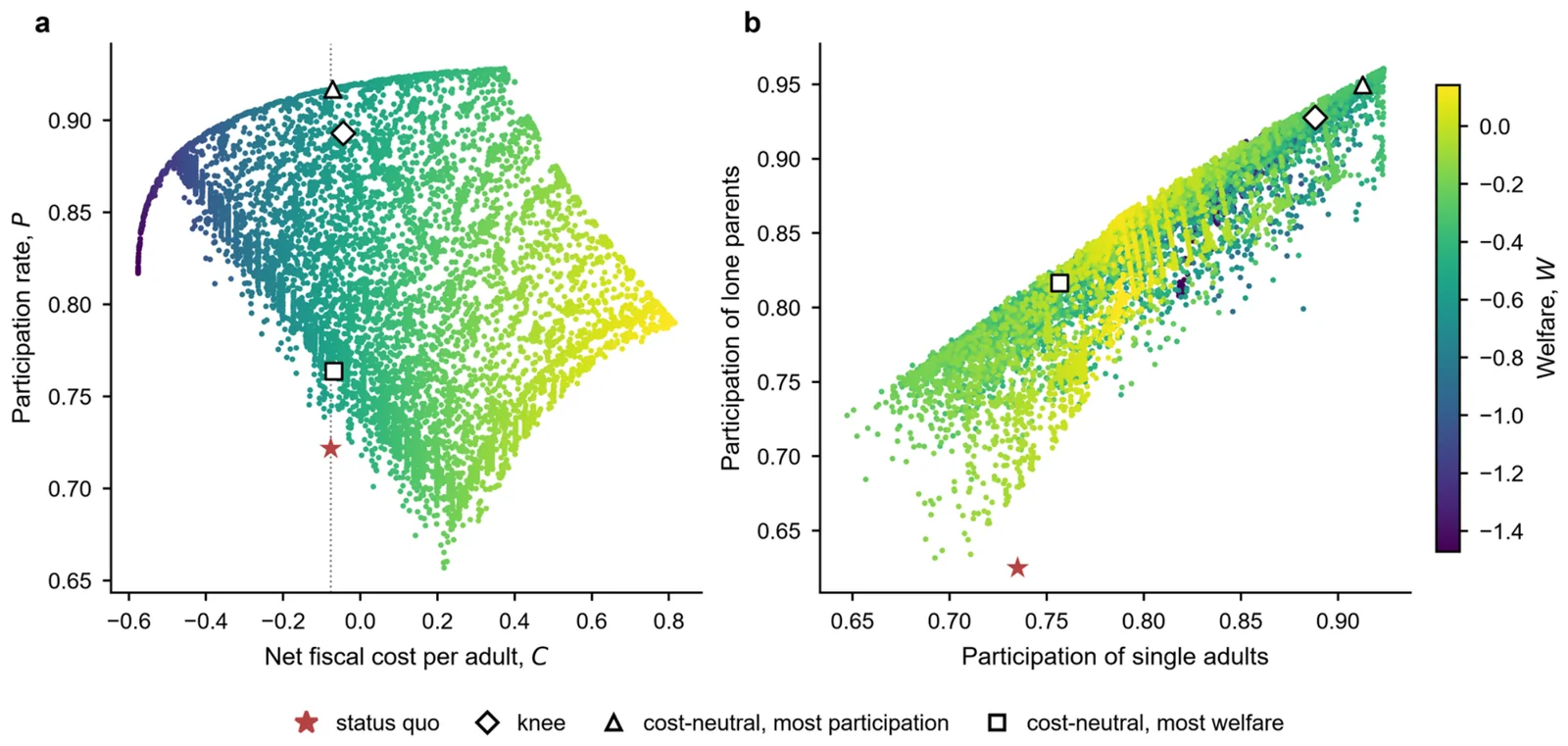
Pooled Pareto set of the parametric-instrument problem: (**a**) participation against cost; (**b**) participation of lone parents against single adults.

On PI, scored against the 10,344 non-dominated points pooled from all runs (10,205 after removing duplicates), AR-MOEA (median 0.0365) again comes closest. The decomposition algorithms and ablation variants follow between 0.0417 and 0.0424, with no significant difference between MOEA/D-DoL and any of them, ahead of NSGA-III (0.0502), NSGA-II (0.0619), RVEA (0.0687), MOPSO (0.1261) and MOGWO (0.1996). By hypervolume, which rewards the extremes of a front, the order is different: AR-MOEA (0.863), NSGA-III (0.848) and NSGA-II (0.842) come first, followed by the decomposition algorithms and ablation variants (0.835–0.836), level with RVEA (0.834), ahead of MOPSO (0.723) and MOGWO (0.592); MOEA/D-DE and MOEA/D-FRRMAB are significantly ahead of MOEA/D-DoL by hypervolume, by a negligible 0.07–0.08%. The pooled reference is densest where its seven decomposition configurations place their points, which favours them under IGD; hypervolume does not depend on the density of a reference sample (the reference front enters only through its ideal and nadir points), and by it AR-MOEA, NSGA-III and NSGA-II lead the decomposition algorithms on PI, as NSGA-II and AR-MOEA do on FS. At the status quo cost, the best schedule of a single run raises participation to 90.7% for AR-MOEA but only to 77.3% for MOEA/D-DoL and 77.0% for MOEA/D-DE (medians over runs), against 91.7% in the pooled set. Figure 9 shows the pooled Pareto set of 10,205 distinct schedules together with the status quo.

The status quo, with a participation rate of 72.2%, is dominated: 4.6% of the Pareto-optimal schedules are better on all three objectives at once. At the status quo cost (the dotted line in Figure 9a), the Pareto set offers participation rates between 74.2% and 91.7%; welfare peaks at a participation rate of 76.4% and falls as participation rises beyond it along that line. Figure 9b shows that most schedules that raise participation raise it for both household types, and those with the highest participation raise it more for lone parents, whose status quo participation is lowest; 5.5% of the schedules that raise overall participation lower that of single adults.

### 6.3. What the Pareto Set Says About Policy

Table 7 compares the status quo with three Pareto-optimal schedules: the one with the highest participation and the one with the highest welfare among those whose net cost is within 0.01 of median earnings per adult of the status quo’s, and the knee of the set. The cost-neutral participation maximum raises participation from 72.2% to 91.7%, and that of lone parents from 62.5% to 95.0%, at a welfare level close to the status quo (−0.635 against −0.641). The cost-neutral welfare maximum raises welfare to −0.532 and participation to 76.4%. The knee reaches 89.3% participation and welfare −0.570 for an additional net cost of 0.032 of median earnings per adult.

**Figure 10:**
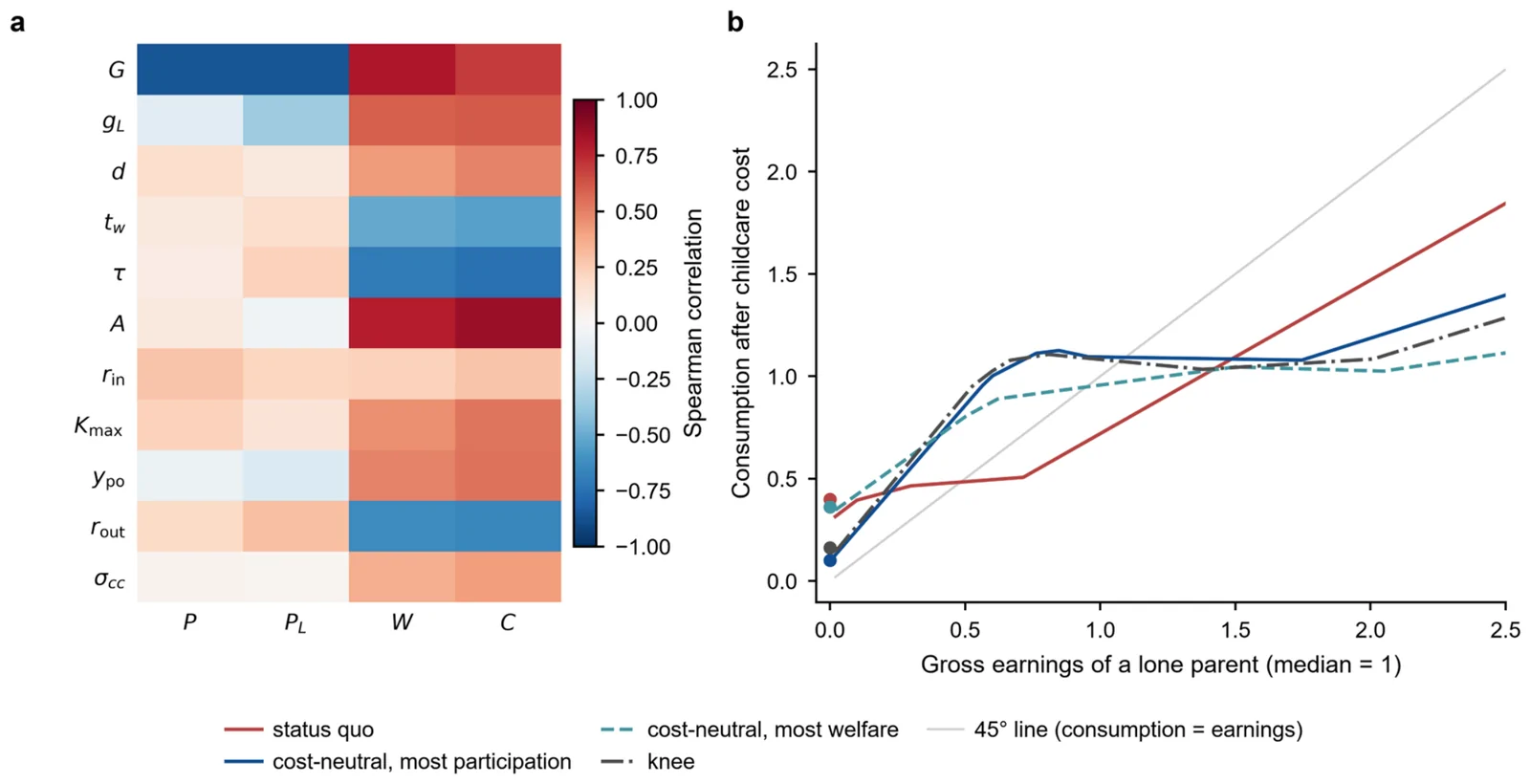
Parametric-instrument Pareto set: (**a**) Spearman correlations of instruments with participation, welfare and cost; (**b**) lone parents’ budget constraints.

The three schedules share a direction of reform. Each withdraws benefit far more slowly than the status quo (taper 0.21–0.26 against 0.65), disregards more earnings (0.51–0.60 against 0.10), pays an in-work credit that the status quo does not have (phase-in rate 0.52–0.60), refunds most of a working lone parent’s childcare cost (0.66–0.97 against 0.30), drops the out-of-work child element, and pays for this with a higher income-tax rate (0.58–0.59 against 0.25). Along the whole Pareto set, the instrument most strongly associated with the trade-off between participation and welfare is the guaranteed income, with a Spearman correlation of −0.86 with participation and 0.81 with welfare (see Figure 10). Section 6.4 shows which of these features survive other calibrations.

Figure 10b shows what this does to a lone parent’s budget; the dots at zero earnings mark her income out of work. Every one of the three schedules lowers her income out of work and raises the reward for the first units of earnings, where the status quo withdraws benefit and taxes earnings at a combined 90% between 0.30 and 0.72 of median earnings. They do not remove high combined withdrawal, however; they move it up the earnings scale, where the credit’s phase-out adds to the taper and the tax: 128% between 0.85 and 0.96 and 102% between 0.96 and 1.75 for the cost-neutral participation maximum, 104% between 1.50 and 2.05 for the cost-neutral welfare maximum and 113% between 0.81 and 1.38 and 92% between 1.38 and 2.01 for the knee. Over the stretches above 100%, consumption falls as earnings rise. The model does not penalise such rates, because hours do not respond in it (Section 7.3). For the same reason, a high tax rate costs little in the model: it distorts no choice of hours and acts on participation only through the gain from work, which the in-work credit restores at the bottom of the earnings scale, so the financing of the credit by a higher tax rate partly reflects the absence of an hours margin. Two checks show what this does to the result (Table A6 in Appendix C), each pooling 30 AR-MOEA runs. Capping the effective marginal rate at 100% for both household types at every earnings level leaves the cost-neutral participation maximum at 91.7% (91.4% without the cap in the same number of runs) and its direction unchanged: a guaranteed income at its floor, a taper of 0.20, a disregard of 0.60, an in-work credit and a tax rate of 0.59, with a highest marginal rate of 95%. Widening the ranges of the ten instruments that can be widened (Appendix C) also preserves the direction, a lower guaranteed income, a slower taper, a larger disregard, an in-work credit and a higher tax rate, and raises the cost-neutral maximum to 94.1%, but the instruments move to or near the new bounds: the guaranteed income and the taper to 0.05, the phase-in rate to 0.98 and the tax rate to 0.80. With the cap as well, the tax rate stops at 0.73 and the maximum credit moves to its new bound of 0.80 instead. The direction agrees with the case for in-work benefits in countries with high benefit phase-out rates [8] and with the optimal earned income credit when responses are concentrated on the extensive margin [21]. The magnitudes deserve less weight: in 54% of the Pareto set the taper lies within 2% of its range from its lower limit of 0.20, in 55% the credit’s phase-in rate lies as close to its upper limit of 0.60, and in 30–44% the disregard, tax rate, maximum credit, childcare refund and phase-out threshold lie as close to their upper limits. The ranges of Table 2, not the model, stop these instruments, and wider ranges mostly move the bounds that bind, so the direction of reform is a result of the analysis, while its size is set by the ranges allowed and, through the cheap tax rate, by the absence of an hours margin.

### 6.4. Sensitivity to the Calibration

Table 8 repeats the read-out under the twelve alternative calibrations of Section 3.4, each changing one parameter, with ten runs per calibration of AR-MOEA, the most accurate algorithm on PI by both IGD and hypervolume (Section 6.2) and the one that covers the largest share of the exact FS front at *t* = 0.05 and at every distance from 0.015 to 0.10 (Section 6.1). So that the rows can be compared, the baseline is pooled from ten AR-MOEA runs in the same way; it reaches a cost-neutral participation rate of 91.0%, against 91.7% for the pool of all runs. Pooling thirty baseline runs instead of ten raises it only to 91.4%. Across three disjoint sets of ten runs, the guaranteed income, the phase-in rate and the tax rate of that schedule vary by less than 0.03, whereas the taper (0.20–0.30), the maximum credit (0.31–0.40) and above all the childcare refund (0.80, 0.98 and 0.03) are not pinned down even at the baseline. Here and in Table 7, a schedule is cost-neutral when its net cost is within 0.01 of median earnings per adult of the status quo’s.

The size of the attainable gain depends on the calibration. The cost-neutral schedule with the highest participation raises it by 18.8 percentage points in the baseline, by as little as 11.9 points under ‘Single adults’ elasticity 0.10’ and by as much as 23.3 under ‘Single adults’ elasticity 0.40’. Its structure does not. In all twelve calibrations, it sets the guaranteed income within 0.02 of its floor of 0.10, pays an in-work credit with a phase-in rate of at least 0.50 and sets the tax rate at 0.55 or more; in eleven it keeps the out-of-work child element at 0.05 or less. In every calibration, its welfare stays within 0.08 of the status quo’s. Other instruments are not pinned down. The taper ranges from 0.20 to 0.41 and the earnings disregard from 0.31 to 0.60, which is to be expected: with out-of-work income near its floor, the benefit they act on is worth only about 0.10–0.15 of median earnings. The childcare refund, part of the common direction in Table 7, ranges from 0.12 to 1.00 and is therefore not a robust feature of the reform. Each row of Table 8 changes one parameter, so we also drew 50 calibrations jointly, by Latin hypercube sampling of the seven parameters over the intervals spanned by their alternative values, with ten AR-MOEA runs each (Table A6 in Appendix C). The attainable gain then varies more, from 9.7 to 23.8 percentage points (median 18.5), mainly with the elasticity of single adults (rank correlation 0.82), and the welfare of the schedule stays within 0.11 of the status quo’s. The direction does not vary: in all 50 draws the cost-neutral schedule with the highest participation lowers the guaranteed income and the taper, raises the disregard and the tax rate and pays an in-work credit; it raises the childcare refund in 47 draws and keeps the child element at 0.05 or less in 38 (but at or above its status quo level of 0.10 in 8), while the guaranteed income sits within 0.02 of its floor in 35 draws and the tax rate reaches 0.55 in 45.

## 7. Discussion

### 7.1. What the Colony Contributes

What does the colony add to MOEA/D? Measured by accuracy, less than the elegance of the correspondence might suggest. On the seventeen benchmarks, MOEA/D-DoL is significantly better than its core by IGD on three problems (DTLZ6 by a negligible margin) and worse on one, with both clear gains on problems used in its design; over the seventeen benchmarks, its mean rank of 4.06 against 4.76 is a difference far smaller than the Nemenyi critical difference, and it stays behind the best Pareto-based and reference-point algorithms by a margin that the Nemenyi test cannot resolve, either; on the nine held-out WFG problems by IGD, it is better than its core on three, one of them negligibly, and worse on none, but ranks only fifth of the nine algorithms; on the policy problems, it leaves IGD unchanged and trails MOEA/D-DE and MOEA/D-FRRMAB negligibly (by 0.07–0.08%) in hypervolume on PI, although on FS its points lie significantly closer to the exact front. What the colony does change is how effort is spent. It produces a division of labour that can be read from a run (Section 5.3), it is ahead of its core on UF1 at a quarter of the budget, and on UF2 and UF7 its median IGD reaches the core’s final median only between 65% and 70%, and between 55% and 60%, of the budget, and it moves the failed runs of the decomposition core from some problems to others. The ablations (Section 5.4) show that every part of the colony is needed for the gains on UF7 and DTLZ6, and every part except threshold learning for the gain on UF2; on most problems no part of the colony makes a detectable difference. For a user these gains are small: on the policy problems, MOEA/D-DoL and its core differ by at most 0.4 percentage points of participation at the status quo cost, whereas the choice of algorithm changes it by 13 to 19 points (AR-MOEA against MOEA/D-DoL: 86.3% against 67.7% on FS and 90.7% against 77.3% on PI; Section 6.1 and Section 6.2).

The failure of the literal rule is itself informative. A fixed increment per success or failure presumes a success rate that stays in one range. In an optimiser the rate of successful offspring falls steadily as the population approaches the front, so a reinforcement rule that is to keep a division of labour alive has to compare a worker with its colony rather than with a fixed standard. Lemma 1 shows that the relative rule does exactly that, and it says what is being learned: which subproblems are currently easier to improve than the average. This is the question that the utility functions of MOEA/D-GRA answer with an engineered formula [29]; the colony answers it with a stimulus that no task can monopolise and a threshold that moves only when a worker works. MOEA/D-FRRMAB, which combines an engineered allocation with a bandit over four differential-evolution operators, is better than MOEA/D-DoL on five problems and worse on three; with both components in one algorithm, these experiments cannot say whether its advantage comes from the allocation or from the operators.

How much any allocation rule can help also depends on the problem. Algorithms built on systematically generated weight vectors perform well on the DTLZ and WFG problems but deteriorate after slight changes of their formulations, because of how the features of these problems relate to the weight vectors [57]. Deciding which subproblems deserve effort cannot repair a mismatch between the weight vectors and the front. The DTLZ suite contains both kinds: fronts that match a simplex of weights (DTLZ1–DTLZ4) and degenerate or disconnected ones (DTLZ5–DTLZ7), on which NSGA-II and AR-MOEA are ahead of every decomposition algorithm. MOEA/D-DoL and MOEA/D-DE differ significantly in IGD on one of the seven (DTLZ6) and in hypervolume on two (DTLZ5 and DTLZ6), each time by a negligible amount. On the policy problems, whose objectives differ in scale, normalising the objectives inside the decomposition lowered the median IGD on FS from 0.137 to 0.054, far more than any allocation rule changed it (Section 6.1).

### 7.2. What the Policy Problems Show

The policy problems put the correspondence of Section 2 to work. Because participation follows the response function, a schedule acts on each adult’s decision only through her stimulus, the utility gain from work, and the Pareto set says which combinations of instruments raise that stimulus where it matters most. The three-way trade-off is real: at no extra cost, the planner can have participation of 92% or welfare of −0.532, but not both, and the guaranteed income out of work is the lever that moves between them. The schedules that come out on top combine in-work support and low withdrawal at the bottom of the earnings distribution, which microsimulation finds desirable where phase-out rates are high [8], with a higher flat tax rate above the allowance; they also move high combined withdrawal further up the earnings scale, which a model with an hours margin would penalise, and a cap of 100% on the marginal rate removes the stretches above 100% (the highest rate becomes 95%) without lowering the cost-neutral participation maximum (Section 6.3). What the evolutionary search adds is the whole menu: it shows the planner every Pareto-optimal compromise instead of a single optimum for one set of welfare weights.

The exact front of FS gives the policy problem a property that applied studies of evolutionary optimisation rarely have: the accuracy of every algorithm on a policy problem, rather than on a synthetic benchmark, is known. The separability that makes FS exactly solvable disappears once the schedule has to be built from a guaranteed income, tapers, allowances and a credit, which is why PI can only be scored against the best that the algorithms find. The two problems agree on the most accurate algorithm (AR-MOEA) and on the least accurate, the two swarm algorithms. By IGD they disagree on the middle, the decomposition algorithms trailing NSGA-II, NSGA-III and RVEA on FS and leading them on PI (rank correlation of the nine median IGD values 0.53); by hypervolume, which does not depend on the density of a reference sample, AR-MOEA and NSGA-II lead the decomposition algorithms on both problems and NSGA-III on PI only. A pooled front measures closeness to the best that was found, and it is densest where the most numerous configurations put their points; only an exact front measures closeness to the truth.

### 7.3. Limitations

The economy is stylised. Its behavioural parameters are anchored to published elasticities, and Section 6.4 varies each of them, but the earnings distribution, the shares of household types and the status quo schedule describe no particular country, and the results are statements about the structure of the trade-offs rather than forecasts for a reform. Only the extensive margin is modelled: adults work or do not, and the hours of those who work do not respond, which is the case in which the correspondence of Proposition 1 is exact and the case in which the evidence of Section 1 locates most of the response. Welfare is utilitarian with logarithmic utility; a planner with a rank-dependent welfare criterion, which trades total income off against inequality measured by the Gini index [58], would rank the same outcomes differently; its preferred schedule need not lie on the (*P*,*W*,*C*) front at all, and finding it would require the front to be recomputed with that criterion in place of *W*. Couples, whose second earners respond differently [11], are left out, as are dynamic effects of work experience on later earnings, the take-up and administration of benefits, and any effect of the schedule on wages or labour demand. Because hours do not respond, the model charges high marginal rates above the entry point only through their effect on the gain from work, not through any loss of hours, which flatters a high tax rate and steep withdrawal in work; with an hours margin these would carry an efficiency cost, and the optimal-tax results with both margins [21,24] make the case for an in-work credit depend on the relative size of the two responses. Which conclusions are therefore likely to travel? The direction of reform, a lower guaranteed income, a slower taper, a larger disregard and an in-work credit financed by a higher tax rate above an allowance, held under every single-parameter calibration and in all 50 joint draws of the calibration parameters, and also when marginal rates are capped at 100% or the instrument ranges are widened, and it is the direction that optimal-tax theory predicts when responses are concentrated on the extensive margin [21]. The magnitudes did not travel: the attainable participation gain varied from 9.7 to 23.8 percentage points across 50 joint calibration draws, and many instruments sit at the bounds of their ranges. A result for a particular country would require its own earnings distribution, household types and status quo schedule.

On the algorithmic side, MOEA/D-DoL was designed with pilot runs on eleven of the seventeen benchmark problems, with seeds disjoint from those of the reported runs, and its parameters were fixed before the reported experiments. On these seventeen problems the comparison is therefore out of sample in its seeds but mostly not in its problems, and its clear gains over MOEA/D-DE are all on pilot problems. The held-out WFG suite is out of sample in both: there, MOEA/D-DoL gains clearly in IGD on two of the nine problems (WFG1 and WFG4), in hypervolume also on WFG8, and loses on none, so its gains are not confined to the problems used in its design, although they remain modest and leave it behind the Pareto-based and reference-point algorithms. Section 5.5 shows that the results do not depend on a finely tuned parameter setting. The benchmarks are continuous and unconstrained, and have at most three objectives. Whether the colony helps in many-objective problems, where the number of subproblems grows quickly and most of them improve rarely, is the most interesting open question, because it is there that a rule that finds the few productive tasks without being told should matter most.

## 8. Conclusions

The study establishes a correspondence and an algorithm built on it. Under four assumptions (a binary decision to work and a private cost of working that is additive in utility, independent of consumption and of the schedule, and log-logistic), the share of adults who work at each earnings level is the insect response function, with the utility gain from work as the stimulus (Proposition 1), and the response exponent follows from the participation elasticity (Corollary 1). MOEA/D-DoL borrows the mechanism of this correspondence and divides the work of a decomposition-based algorithm among threshold-driven workers. A literal transfer of the ants’ reinforcement rule fails in an optimiser, and comparing each worker with its colony is what keeps the division of labour alive: it forms in every one of 30 runs on UF9, and in all 210 traced runs, a worker’s relative success predicts its later engagement. As an optimiser the colony adds little. By IGD, MOEA/D-DoL is clearly better than its core on two of the seventeen main benchmarks, UF2 and UF7 (median IGD 16% and 29% lower), both used in its design, negligibly better on a third, DTLZ6 (0.055%), and worse on one, UF3; on the nine held-out WFG problems, it is clearly better on two, WFG1 and WFG4 (9.5% and 1.1% lower), negligibly better on a third, WFG5 (0.2%), and worse on none. It ranks fourth of the nine algorithms on the seventeen benchmarks, behind NSGA-III, AR-MOEA and NSGA-II, and fifth on the WFG problems, where RVEA also ranks higher, although the Nemenyi test separates none of these algorithms from it. No parameter setting tried in Section 5.5 changed its median IGD significantly.

For policy design, the exact supported front of the free-schedule problem proved more informative than any benchmark. It showed that decomposition algorithms, ours included, cover only a quarter of the front of a badly scaled policy problem, so that a planner relying on one of their runs would miss participation gains available at no extra cost; AR-MOEA, which normalises the objectives, comes much closer. At the status quo cost, the schedules with the highest participation share one direction of reform: a lower guaranteed income, a slower benefit taper, a larger earnings disregard and an in-work credit, paid for by a higher tax rate above an allowance. None of the robustness checks of Section 6.3 and Section 6.4 (twelve single-parameter calibrations, 50 joint calibration draws, marginal rates capped at 100% and widened instrument ranges) reversed this direction, whereas the gain itself varied from 9.7 to 23.8 percentage points across the joint draws.

Several things remain uncertain. The correspondence is exact only for the extensive margin with a log-logistic cost, although the log-normal and Weibull costs we tried come within 2 and 6 percentage points of it. The policy model has no hours margin, no couples and no take-up, administration or labour-demand effects, so the low cost of financing the credit by a higher tax rate partly reflects the model, and the magnitudes are specific to a stylised calibration and to instrument ranges, several of which bind. On the algorithmic side, the gains of the colony are small and uneven across problems. We would give priority to three directions: adding an hours margin and couples to the policy model and calibrating it to the earnings distribution and schedule of a particular country; combining the colony with objective normalisation, which closes most of the accuracy gap of decomposition on badly scaled problems; and testing whether it helps on many-objective problems, the open question raised in Section 7.3.

## Figures and Tables

**Table 1:** Correspondence between division of labour, labour-force participation and the proposed algorithm.

Colony (Fixed-Threshold Model)	Labour Market (Proposition 1)	MOEA/D-DoL (Section 4)
Worker	Adult at earnings level *i*	Individual *j* of the population
Task	Paid work	Improving subproblem *j*
Stimulus *s*	Utility gain from work *D_i_*	Demand that builds up while subproblem *j* waits, *s_j_*
Threshold *θ*	Scale *a_i_* of the cost of work	Threshold *θ_j_*
Response exponent *n*	Shape of the cost distribution, implied by the assumed elasticity	*n* = 2, a design constant (Section 5.5)
Probability of engaging *T_θ_*(*s*)	Participation rate *P_i_*	Probability of producing an offspring
Threshold reinforcement	Not modelled	*θ_j_* falls with above-average success, rises otherwise

**Table 2:** The eleven instruments of the parametric-instrument problem, their ranges and the status quo.

Symbol	Instrument	Range	Status Quo
*G*	Guaranteed income of an adult out of work	[0.10, 0.60]	0.30
*g_L_*	Child element added for a lone parent	[0.00, 0.30]	0.10
*d*	Earnings disregard before the benefit taper starts	[0.00, 0.60]	0.10
*t_w_*	Benefit taper (withdrawal) rate	[0.20, 1.00]	0.65
*τ*	Income tax rate	[0.10, 0.60]	0.25
*A*	Income tax allowance	[0.00, 1.20]	0.30
*r*_in_	Phase-in rate of the in-work credit	[0.00, 0.60]	0.00
*K*_max_	Maximum in-work credit	[0.00, 0.40]	0.00
*y*_po_	Earnings at which the credit starts to phase out	[0.20, 1.50]	0.80
*r*_out_	Phase-out rate of the in-work credit	[0.05, 0.50]	0.20
*σ_cc_*	Share of the childcare cost refunded to a working lone parent	[0.00, 1.00]	0.30

**Table 3:** Calibration of the participation economy.

Parameter	Symbol	Value	Basis
Earnings levels	*I*	20	Mid-quantiles of a log-normal distribution with median 1
Dispersion of log earnings	*σ*	0.6	Stylised; varied in Section 6.4
Share of lone parents	*π_L_*	0.12	Stylised; varied in Section 6.4
Childcare cost of a working lone parent	*κ*	0.15	Share of median earnings; varied in Section 6.4
Status quo participation, single adults	$F_{S,i}^{0}$	0.55–0.92	Rising linearly across earnings levels; varied in Section 6.4
Status quo participation, lone parents	$F_{L,i}^{0}$	0.40–0.85	Rising linearly across earnings levels; varied in Section 6.4
Mean participation elasticity, single adults	${\bar{\eta }}_{S}$	0.25	Small elasticities [17]; varied in Section 6.4
Mean participation elasticity, lone parents	${\bar{\eta }}_{L}$	0.50	Stronger response of lone mothers [18]; varied in Section 6.4
Response exponent, single adults	*n_S_*	1.38	Corollary 1
Response exponent, lone parents	*n_L_*	1.67	Corollary 1
Threshold (scale of the cost of work), single adults	*a_S_*_,*i*_	0.36–0.48	Proposition 1 at the status quo
Threshold (scale of the cost of work), lone parents	*a_L_*_,*i*_	0.19–0.64	Proposition 1 at the status quo

Elasticities are with respect to the financial gain from work, averaged over earnings levels with population weights; weighted by participation instead, they are 0.23 and 0.44. The free-schedule problem uses the single-adult calibration with a status quo of *c*_0_ = 0.30 and *c_i_* = 0.30 + 0.70 *w_i_*, which gives *n* = 1.52. At the status quo of Table 2, the parametric economy has a participation rate of 72.2% (73.5% of single adults and 62.5% of lone parents) and a net fiscal cost of −0.077 per adult, a net revenue.

**Table 4:** Comparison algorithms and their parameter settings.

Algorithm	Family	Parameter Settings
MOEA/D-DoL	Decomposition with a colony	Core as MOEA/D-DE; colony parameters as in Section 4.2
MOEA/D-DE	Decomposition	*H* = 20, *p*_nb_ = 0.9, *n_r_* = 2, *CR* = 1, *F* = 0.5; polynomial mutation with *η_m_* = 20 and rate 1/*n_x_*
MOEA/D-FRRMAB	Decomposition with operator bandit	Core as MOEA/D-DE with four DE operators; scaling factor *C* = 5, decay factor *D* = 1, sliding window of *N*/2
NSGA-II	Pareto dominance	SBX with *η_c_* = 20 and rate 1; polynomial mutation with *η_m_* = 20 and rate 1/*n_x_*
NSGA-III	Reference points	SBX with *η_c_* = 30 and rate 1; polynomial mutation with *η_m_* = 20 and rate 1/*n_x_*
RVEA	Reference vectors	*α* = 2, *f_r_* = 0.1; SBX with *η_c_* = 30; polynomial mutation with *η_m_* = 20
AR-MOEA	Adaptive reference points	SBX with *η_c_* = 20; polynomial mutation with *η_m_* = 20
MOPSO	Particle swarm	Inertia weight 0.4; 10 grid divisions per objective; repository of size *N*
MOGWO	Grey wolf swarm	10 grid divisions, inflation *α* = 0.1, leader pressure *β* = 4, deletion pressure *γ* = 2; archive of size *N*

*n_x_* is the number of decision variables and *N* the population size. Settings are those of the original publications or of their PlatEMO implementations; none of the comparison algorithms was tuned on the test problems, whereas the colony parameters of MOEA/D-DoL were chosen in pilot runs on eleven of them (Section 4.2 and Appendix B); population size, evaluation budget and stopping rule are common to all algorithms.

**Table 5:** Ablation of MOEA/D-DoL: variants and their comparison with the full algorithm on the seventeen benchmark problems.

Variant	What Is Removed	Mean Rank	Better/Equal/Worse
MOEA/D-DoL	Nothing: the full algorithm	1.94	—
DoL-fixed	Threshold learning: every threshold stays at *θ*_0_	2.65	0/15/2
DoL-abs	$\mathrm{The}\;\mathrm{comparison}\;\mathrm{with}\;\mathrm{the}\;\mathrm{colony}:\;\mathrm{failure}\;\mathrm{raises}\;\mathrm{the}\;\mathrm{threshold}\;\mathrm{by}\;\mathrm{the}\;\mathrm{constant}\;\xi {\bar{r}}_{0}/(1-{\bar{r}}_{0})$	3.12	0/14/3
DoL-random	The choice of workers: each works with the constant probability *q*	3.88	0/14/3
DoL-all	The division of labour: every worker works in every generation, in random order	3.41	0/14/3

Mean rank of median IGD among the variants over the seventeen problems (lower is better); medians are used because a few failed runs dominate the means (Section 5.2). The last column counts the problems on which the variant is significantly better than, not significantly different from, or significantly worse than MOEA/D-DoL (Wilcoxon rank-sum test, Holm-corrected over the four variants, *α* = 0.05).

**Table 6:** Normalised IGD and hypervolume on the two policy problems (median and interquartile range over 30 runs).

Algorithm	FS IGD	FS HV	PI IGD	PI HV
MOEA/D-DoL	1.37e-1 (3.7e-3)	8.82e-1 (2.3e-3)	4.21e-2 (1.2e-3)	8.35e-1 (1.2e-3)
MOEA/D-DE	1.37e-1 (3.3e-3) ≈	8.82e-1 (1.9e-3) ≈	4.21e-2 (7.9e-4) ≈	8.36e-1 (1.4e-3) −
MOEA/D-FRRMAB	1.35e-1 (5.5e-3) ≈	8.83e-1 (3.0e-3) ≈	4.19e-2 (9.1e-4) ≈	8.36e-1 (9.2e-4) −
NSGA-II	6.08e-2 (9.0e-3) −	**8.98e-1 (7.5e-3) −**	6.19e-2 (8.8e-3) +	8.42e-1 (6.1e-3) −
NSGA-III	6.70e-2 (1.1e-2) −	8.56e-1 (1.3e-2) +	5.02e-2 (4.2e-3) +	8.48e-1 (3.8e-3) −
RVEA	1.05e-1 (2.4e-2) −	8.55e-1 (1.0e-2) +	6.87e-2 (5.6e-3) +	8.34e-1 (6.5e-3) ≈
AR-MOEA	**5.16e-2 (3.6e-3) −**	8.94e-1 (1.2e-2) −	**3.65e-2 (1.1e-3) −**	**8.63e-1 (1.9e-3) −**
MOPSO	1.94e-1 (1.6e-2) +	6.13e-1 (1.7e-2) +	1.26e-1 (9.1e-3) +	7.23e-1 (1.7e-2) +
MOGWO	2.64e-1 (3.5e-2) +	5.97e-1 (7.3e-2) +	2.00e-1 (3.2e-2) +	5.92e-1 (5.0e-2) +
DoL-fixed	1.37e-1 (2.1e-3) ≈	8.82e-1 (1.2e-3) ≈	4.22e-2 (6.3e-4) ≈	8.35e-1 (1.0e-3) ≈
DoL-abs	1.37e-1 (3.1e-3) ≈	8.82e-1 (2.2e-3) ≈	4.17e-2 (6.7e-4) ≈	8.35e-1 (1.1e-3) ≈
DoL-random	1.38e-1 (2.3e-3) ≈	8.81e-1 (1.2e-3) ≈	4.21e-2 (7.2e-4) ≈	8.35e-1 (7.4e-4) ≈
DoL-all	1.36e-1 (2.9e-3) ≈	8.82e-1 (2.2e-3) ≈	4.24e-2 (8.0e-4) ≈	8.35e-1 (1.4e-3) ≈

Objectives are scaled by the ideal and nadir points of the reference front: the exact front for FS and the pooled non-dominated set of all runs for PI. The best median is in bold. + (−) marks an algorithm significantly worse (better) than MOEA/D-DoL and ≈ no significant difference (Wilcoxon rank-sum test, *α* = 0.05, Holm-corrected separately over the comparison algorithms and over the ablation variants, for IGD and for HV).

**Table 7:** Status quo and three representative Pareto-optimal policies of the parametric-instrument problem.

	Status Quo	Cost-Neutral, Most Participation	Cost-Neutral, Most Welfare	Knee
*G* guaranteed income of an adult out of work	0.30	0.10	0.36	0.16
*g_L_* child element added for a lone parent	0.10	0.00	0.00	0.00
*d* earnings disregard before the benefit taper starts	0.10	0.56	0.51	0.60
*t_w_* benefit taper (withdrawal) rate	0.65	0.26	0.23	0.21
*τ* income tax rate	0.25	0.58	0.59	0.59
*A* income tax allowance	0.30	0.60	0.00	0.53
*r*_in_ phase-in rate of the in-work credit	0.00	0.52	0.54	0.60
*K*_max_ maximum in-work credit	0.00	0.40	0.33	0.40
*y*_po_ earnings at which the credit starts to phase out	0.80	0.85	1.50	0.81
*r*_out_ phase-out rate of the in-work credit	0.20	0.44	0.21	0.33
*σ_cc_* share of the childcare cost refunded to a working lone parent	0.30	0.97	0.77	0.66
Participation rate	72.2%	91.7%	76.4%	89.3%
Participation, single adults	73.5%	91.3%	75.7%	88.8%
Participation, lone parents	62.5%	95.0%	81.6%	92.8%
Welfare	−0.641	−0.635	−0.532	−0.570
Net fiscal cost per adult	−0.077	−0.072	−0.069	−0.045

Amounts in units of median earnings. The knee is the Pareto-optimal policy closest to the ideal point after each objective is scaled to [0, 1].

**Table 8:** Pareto sets of the parametric-instrument problem under alternative calibrations.

Calibration	Status Quo *P*	Share Dominating Status Quo	Cost-Neutral Max *P*	*P_L_* at that Schedule	Change in *W*	*G*	*r*_in_
Baseline	72.2%	7%	91.0%	94.5%	+0.014	0.12	0.58
Single adults’ elasticity 0.10	72.2%	5%	84.0%	94.6%	−0.080	0.10	0.59
Single adults’ elasticity 0.40	72.2%	5%	95.5%	95.0%	+0.061	0.10	0.60
Lone parents’ elasticity 0.25	72.2%	6%	89.9%	84.4%	+0.000	0.11	0.60
Lone parents’ elasticity 0.75	72.2%	7%	91.9%	97.1%	+0.007	0.10	0.58
Childcare cost 0.10	72.2%	6%	91.5%	93.5%	−0.007	0.10	0.60
Childcare cost 0.20	72.2%	6%	91.5%	94.0%	−0.002	0.10	0.53
Earnings dispersion 0.4	72.2%	3%	91.5%	95.0%	−0.023	0.10	0.57
Earnings dispersion 0.8	72.2%	11%	91.9%	94.8%	+0.031	0.10	0.60
Share of lone parents 0.06	72.8%	6%	91.0%	94.4%	+0.002	0.11	0.60
Share of lone parents 0.20	71.3%	7%	91.6%	94.8%	+0.000	0.11	0.59
Singles’ participation 0.50–0.88	68.2%	5%	88.2%	94.6%	−0.039	0.10	0.58
Lone parents’ participation 0.30–0.80	71.3%	5%	90.6%	90.1%	+0.000	0.11	0.60

Each row changes one parameter of Table 3. The last five columns describe the Pareto-optimal schedule with the highest participation rate among those whose net cost is within 0.01 of the status quo’s; the change in welfare is relative to the status quo. Every Pareto set, the baseline included, pools the final populations of ten AR-MOEA runs. All eleven instruments of these schedules are listed in Table A5. Gains are computed from unrounded participation rates.

## Data Availability

The original contributions presented in this study are included in the article. Further inquiries can be directed to the corresponding author. The policy-design problems are calibrated from published estimates cited at the point of use; no individual-level data were used. The comparison algorithms were checked against the PlatEMO platform (https://github.com/BIMK/PlatEMO, accessed on 10 October 2026).

## Abbreviations

The following abbreviations are used in this manuscript: DoL, division of labour; EMTR, effective marginal tax rate; FS, free-schedule problem; HV, hypervolume; IGD, inverted generational distance; MOEA/D, multi-objective evolutionary algorithm based on decomposition; PI, parametric-instrument problem; WFG, Walking Fish Group test suite.

## Appendix A

Table A1 and Table A2 give the statistics behind Section 5.2: the median and interquartile range over 30 runs of IGD and of normalised hypervolume for every algorithm on every benchmark problem, with the outcome of the Wilcoxon test against MOEA/D-DoL for each indicator. Table A3 gives the IGD statistics for the held-out WFG problems of Section 5.6.

**Table A1:** IGD of MOEA/D-DoL and the comparison algorithms on the benchmark problems.

Problem	DoL	DE	FRRMAB	NSGA-II	NSGA-III	RVEA	AR-MOEA	MOPSO	MOGWO
UF1	**5.85e-3 (5.1e-4)**	5.86e-3 (5.0e-4) ≈	7.63e-3 (6.4e-4) +	8.47e-2 (1.9e-2) +	9.24e-2 (1.6e-2) +	8.73e-2 (1.8e-2) +	1.01e-1 (2.1e-2) +	1.64e-1 (7.7e-2) +	1.13e-1 (2.0e-2) +
UF2	**1.16e-2 (1.4e-3)**	1.38e-2 (3.0e-3) +	1.18e-2 (1.1e-3) ≈	3.31e-2 (1.0e-2) +	3.30e-2 (1.3e-2) +	6.02e-2 (6.4e-3) +	3.30e-2 (9.9e-3) +	5.71e-2 (1.8e-2) +	6.93e-2 (2.0e-2) +
UF3	1.34e-2 (1.5e-2)	8.62e-3 (1.0e-2) −	**5.48e-3 (3.0e-3) −**	2.44e-1 (9.9e-2) +	2.47e-1 (7.5e-2) +	3.19e-1 (5.9e-3) +	2.90e-1 (6.1e-2) +	3.08e-1 (2.5e-2) +	5.02e-1 (8.2e-2) +
UF4	7.10e-2 (1.0e-2)	7.21e-2 (9.4e-3) ≈	5.91e-2 (7.8e-3) −	4.47e-2 (5.8e-4) −	**4.22e-2 (6.7e-4) −**	7.49e-2 (1.0e-2) ≈	**4.22e-2 (8.9e-4) −**	9.37e-2 (1.9e-2) +	1.32e-1 (1.3e-2) +
UF5	4.06e-1 (2.6e-1)	3.52e-1 (1.4e-1) ≈	2.91e-1 (1.2e-1) −	2.69e-1 (7.5e-2) −	**2.34e-1 (3.9e-2) −**	2.48e-1 (5.6e-2) −	2.84e-1 (1.0e-1) −	1.59e+0 (3.9e-1) +	1.48e+0 (1.1e+0) +
UF6	2.69e-1 (3.3e-1)	3.10e-1 (3.7e-1) ≈	1.81e-1 (2.4e-1) ≈	1.30e-1 (2.1e-2) ≈	**1.25e-1 (1.8e-2) ≈**	1.34e-1 (1.5e-1) ≈	1.30e-1 (1.6e-2) ≈	7.43e-1 (2.1e-1) +	3.24e-1 (1.9e-1) ≈
UF7	**5.83e-3 (3.2e-3)**	8.27e-3 (2.6e-3) +	7.04e-3 (1.2e-3) +	4.00e-2 (2.0e-1) +	4.96e-2 (2.0e-1) +	2.18e-1 (2.9e-1) +	5.02e-2 (2.7e-1) +	1.62e-1 (2.6e-1) +	7.95e-2 (2.4e-1) +
UF8	**1.28e-1 (2.2e-2)**	1.32e-1 (1.9e-2) ≈	1.40e-1 (1.7e-2) ≈	3.13e-1 (1.8e-1) +	2.69e-1 (6.3e-3) +	3.57e-1 (2.1e-2) +	2.59e-1 (3.5e-3) +	2.74e-1 (1.3e-2) +	3.67e+0 (1.4e+0) +
UF9	2.26e-1 (7.6e-3)	2.30e-1 (1.8e-2) ≈	2.26e-1 (1.5e-2) ≈	3.55e-1 (1.4e-1) +	**1.62e-1 (1.5e-1) ≈**	2.66e-1 (1.6e-1) ≈	2.45e-1 (1.3e-1) ≈	5.04e-1 (9.9e-2) +	6.14e-1 (2.5e-1) +
UF10	5.29e-1 (8.0e-2)	5.25e-1 (1.1e-1) ≈	6.34e-1 (1.7e-1) +	5.64e-1 (1.1e-1) ≈	**3.19e-1 (4.1e-2) −**	5.83e-1 (1.3e-1) ≈	3.44e-1 (2.8e-1) ≈	7.64e-1 (1.4e-1) +	8.46e-1 (1.0e+0) +
DTLZ1	2.83e-2 (1.0e-4)	2.83e-2 (7.6e-5) ≈	2.82e-2 (1.4e-4) −	2.63e-2 (1.5e-3) −	**1.89e-2 (1.6e-5) −**	**1.89e-2 (1.4e-5) −**	**1.89e-2 (1.2e-5) −**	1.18e+0 (9.1e-1) +	1.25e+1 (4.3e+0) +
DTLZ2	6.91e-2 (2.6e-4)	6.92e-2 (1.9e-4) ≈	6.92e-2 (4.6e-4) ≈	6.72e-2 (4.4e-3) −	**5.01e-2 (7.0e-7) −**	**5.01e-2 (7.3e-7) −**	**5.01e-2 (1.1e-5) −**	7.26e-2 (7.8e-3) +	3.36e-1 (3.2e-2) +
DTLZ3	6.99e-2 (1.1e-3)	6.98e-2 (1.3e-3) ≈	6.97e-2 (2.8e-3) ≈	6.79e-2 (3.7e-3) −	5.03e-2 (3.4e-4) −	**5.02e-2 (2.3e-4) −**	5.05e-2 (5.8e-4) −	2.50e+1 (6.6e+1) +	1.75e+2 (2.2e+1) +
DTLZ4	7.30e-2 (3.6e-3)	7.31e-2 (5.3e-3) ≈	7.34e-2 (2.1e-3) ≈	6.69e-2 (3.3e-3) −	**5.01e-2 (5.6e-5) −**	**5.01e-2 (6.6e-7) −**	2.95e-1 (4.9e-1) ≈	9.04e-2 (1.6e-2) +	8.21e-2 (2.1e-2) +
DTLZ5	1.19e-2 (2.8e-5)	1.19e-2 (2.7e-5) ≈	1.18e-2 (9.9e-5) −	5.41e-3 (2.8e-4) −	1.30e-2 (2.3e-3) +	6.23e-2 (1.7e-3) +	**4.66e-3 (1.1e-4) −**	7.97e-3 (9.9e-4) −	1.94e-2 (4.6e-3) +
DTLZ6	1.19e-2 (4.6e-6)	1.19e-2 (7.9e-6) +	1.19e-2 (1.7e-5) ≈	5.49e-3 (3.4e-4) −	1.85e-2 (2.5e-3) +	6.48e-2 (7.6e-3) +	**4.26e-3 (6.1e-5) −**	3.63e+0 (1.8e+0) +	1.40e-2 (2.4e-3) +
DTLZ7	1.81e-1 (1.5e-2)	1.94e-1 (1.5e-2) ≈	1.93e-1 (1.2e-2) ≈	7.33e-2 (3.7e-3) −	6.98e-2 (4.6e-3) −	1.01e-1 (5.1e-3) −	**6.24e-2 (1.7e-3) −**	1.53e+0 (5.9e-1) +	9.21e-2 (1.6e-2) −
+/≈/−		3/13/1	3/9/5	6/2/9	7/2/8	7/4/6	5/4/8	16/0/1	15/1/1

Median (interquartile range) over 30 runs; the best median on each problem is in bold. +, ≈ and − mark comparison algorithms that are significantly worse than, not significantly different from, or significantly better than MOEA/D-DoL (two-sided Wilcoxon rank-sum test of the 30 final values, Holm-corrected over the eight comparisons on each problem, α = 0.05; direction from the medians). DoL = MOEA/D-DoL, DE = MOEA/D-DE, FRRMAB = MOEA/D-FRRMAB.

**Table A2:** Normalised hypervolume of MOEA/D-DoL and the comparison algorithms on the benchmark problems.

Problem	DoL	DE	FRRMAB	NSGA-II	NSGA-III	RVEA	AR-MOEA	MOPSO	MOGWO
UF1	**8.65e-1 (1.2e-3)**	**8.65e-1 (1.2e-3) ≈**	8.61e-1 (1.2e-3) +	7.48e-1 (4.4e-2) +	7.40e-1 (1.9e-2) +	7.28e-1 (5.2e-2) +	7.30e-1 (4.6e-2) +	5.79e-1 (1.3e-1) +	6.82e-1 (7.2e-2) +
UF2	**8.57e-1 (2.6e-3)**	8.54e-1 (4.4e-3) +	8.56e-1 (1.5e-3) +	8.30e-1 (9.0e-3) +	8.27e-1 (1.2e-2) +	7.87e-1 (1.2e-2) +	8.28e-1 (8.8e-3) +	7.89e-1 (2.4e-2) +	7.66e-1 (2.7e-2) +
UF3	8.50e-1 (2.5e-2)	8.58e-1 (2.3e-2) ≈	**8.66e-1 (8.2e-3) −**	5.36e-1 (1.1e-1) +	5.28e-1 (8.0e-2) +	4.82e-1 (3.2e-2) +	4.87e-1 (5.6e-2) +	4.09e-1 (5.1e-2) +	2.34e-1 (7.3e-2) +
UF4	4.21e-1 (1.3e-2)	4.18e-1 (1.6e-2) ≈	4.38e-1 (1.4e-2) −	4.65e-1 (1.4e-3) −	**4.71e-1 (1.1e-3) −**	4.14e-1 (1.6e-2) +	**4.71e-1 (1.7e-3) −**	3.77e-1 (3.1e-2) +	3.13e-1 (1.7e-2) +
UF5	1.65e-1 (1.6e-1)	2.65e-1 (1.8e-1) ≈	1.82e-1 (1.9e-1) ≈	3.20e-1 (6.0e-2) −	**3.28e-1 (7.8e-2) −**	2.89e-1 (8.1e-2) −	3.13e-1 (1.1e-1) −	0.00e+0 (0.0e+0) +	0.00e+0 (0.0e+0) +
UF6	3.33e-1 (1.4e-1)	3.40e-1 (2.7e-1) ≈	3.74e-1 (2.4e-1) ≈	**4.19e-1 (5.0e-2) −**	4.17e-1 (5.6e-2) −	4.03e-1 (1.1e-1) ≈	4.14e-1 (2.9e-2) −	4.09e-4 (3.2e-2) +	1.97e-1 (1.9e-1) +
UF7	**6.99e-1 (6.7e-3)**	6.94e-1 (5.5e-3) +	6.96e-1 (2.2e-3) +	6.43e-1 (1.8e-1) +	6.31e-1 (2.1e-1) +	4.61e-1 (2.5e-1) +	6.28e-1 (2.5e-1) +	4.39e-1 (2.4e-1) +	5.66e-1 (2.2e-1) +
UF8	**5.47e-1 (1.2e-2)**	5.44e-1 (4.9e-2) ≈	5.40e-1 (1.6e-2) ≈	3.28e-1 (1.9e-1) +	4.38e-1 (4.2e-3) +	4.19e-1 (1.4e-2) +	4.39e-1 (2.0e-3) +	3.69e-1 (2.5e-2) +	0.00e+0 (0.0e+0) +
UF9	7.70e-1 (2.0e-2)	7.77e-1 (1.5e-2) ≈	7.73e-1 (1.5e-2) ≈	5.24e-1 (1.6e-1) +	**7.91e-1 (1.3e-1) ≈**	6.45e-1 (2.0e-1) +	7.47e-1 (1.6e-1) ≈	3.27e-1 (1.2e-1) +	2.15e-1 (2.8e-1) +
UF10	1.50e-1 (4.1e-2)	1.45e-1 (5.0e-2) ≈	7.31e-2 (8.5e-2) +	6.88e-2 (6.3e-2) +	**2.26e-1 (1.7e-1) −**	1.56e-1 (9.7e-2) ≈	2.14e-1 (9.6e-2) −	2.12e-2 (2.8e-2) +	3.05e-5 (1.3e-1) +
DTLZ1	1.08e+0 (7.5e-4)	1.08e+0 (9.5e-4) ≈	1.08e+0 (8.7e-4) ≈	1.10e+0 (5.8e-3) −	**1.12e+0 (3.0e-4) −**	**1.12e+0 (3.0e-4) −**	**1.12e+0 (2.2e-4) −**	0.00e+0 (0.0e+0) +	0.00e+0 (0.0e+0) +
DTLZ2	7.07e-1 (1.5e-3)	7.06e-1 (5.7e-4) ≈	7.08e-1 (2.7e-3) ≈	7.10e-1 (6.0e-3) −	**7.49e-1 (3.5e-6) −**	**7.49e-1 (1.1e-5) −**	**7.49e-1 (2.7e-5) −**	6.86e-1 (1.2e-2) +	3.28e-1 (4.0e-2) +
DTLZ3	7.02e-1 (6.5e-3)	7.04e-1 (9.2e-3) ≈	7.04e-1 (1.1e-2) ≈	7.09e-1 (9.7e-3) −	7.44e-1 (5.3e-3) −	**7.46e-1 (4.0e-3) −**	7.44e-1 (6.1e-3) −	0.00e+0 (0.0e+0) +	0.00e+0 (0.0e+0) +
DTLZ4	7.15e-1 (3.6e-3)	7.13e-1 (6.4e-3) ≈	7.16e-1 (3.3e-3) ≈	7.12e-1 (6.1e-3) +	**7.49e-1 (3.7e-4) −**	**7.49e-1 (2.1e-5) −**	6.06e-1 (2.9e-1) ≈	6.17e-1 (3.5e-2) +	6.50e-1 (2.8e-2) +
DTLZ5	2.61e-1 (2.5e-5)	2.61e-1 (3.4e-5) +	2.61e-1 (7.6e-5) +	2.65e-1 (3.6e-4) −	2.59e-1 (2.3e-3) +	2.18e-1 (5.4e-3) +	**2.66e-1 (1.4e-4) −**	2.62e-1 (1.3e-3) −	2.48e-1 (9.4e-3) +
DTLZ6	2.61e-1 (8.3e-6)	2.61e-1 (5.2e-6) +	2.61e-1 (8.0e-6) ≈	**2.66e-1 (2.5e-4) −**	2.55e-1 (1.9e-3) +	2.11e-1 (1.1e-2) +	**2.66e-1 (1.1e-4) −**	0.00e+0 (0.0e+0) +	2.57e-1 (7.0e-3) +
DTLZ7	4.59e-1 (4.7e-3)	4.57e-1 (3.4e-3) ≈	4.58e-1 (3.0e-3) ≈	5.38e-1 (5.6e-3) −	5.44e-1 (2.6e-3) −	5.23e-1 (7.7e-3) −	**5.61e-1 (2.1e-3) −**	5.95e-3 (2.0e-2) +	5.00e-1 (1.3e-2) −
+/≈/−		4/13/0	5/10/2	8/0/9	7/1/9	9/2/6	5/2/10	16/0/1	16/0/1

Median (interquartile range) over 30 runs of the hypervolume after normalisation by the true front, reference point 1.1; higher is better; the best median on each problem is in bold; +, ≈ and − mark comparison algorithms that are significantly worse than, not significantly different from, or significantly better than MOEA/D-DoL (two-sided Wilcoxon rank-sum test of the 30 final hypervolume values, Holm-corrected over the eight comparisons on each problem, α = 0.05; direction from the medians).

**Table A3:** IGD of MOEA/D-DoL and the comparison algorithms on the held-out WFG problems.

Problem	DoL	DE	FRRMAB	NSGA-II	NSGA-III	RVEA	AR-MOEA	MOPSO	MOGWO
WFG1	1.02e+0 (1.7e-1)	1.13e+0 (1.2e-1) +	1.31e+0 (1.0e-1) +	2.14e-1 (1.0e-2) −	1.33e-1 (7.5e-3) −	2.40e-1 (7.2e-2) −	**1.30e-1 (6.4e-3) −**	1.58e+0 (1.0e-1) +	1.66e+0 (8.9e-2) +
WFG2	3.13e-1 (1.4e-2)	3.10e-1 (2.4e-2) ≈	3.20e-1 (9.4e-3) ≈	2.38e-1 (2.8e-1) ≈	4.74e-1 (3.2e-1) ≈	**1.75e-1 (3.2e-1) ≈**	4.73e-1 (3.2e-1) ≈	2.39e-1 (3.4e-1) ≈	2.74e-1 (6.7e-2) −
WFG3	1.82e-1 (5.6e-3)	1.81e-1 (6.1e-3) ≈	1.81e-1 (8.5e-3) ≈	1.86e-1 (2.0e-2) ≈	1.98e-1 (1.5e-1) ≈	2.22e-1 (1.3e-2) +	**1.12e-1 (4.1e-3) −**	6.78e-1 (4.0e-2) +	4.73e-1 (8.1e-2) +
WFG4	3.54e-1 (7.6e-3)	3.58e-1 (6.8e-3) +	3.56e-1 (9.4e-3) ≈	2.64e-1 (1.3e-2) −	**2.04e-1 (3.3e-4) −**	2.11e-1 (4.5e-3) −	**2.04e-1 (7.8e-4) −**	8.28e-1 (1.0e-1) +	1.21e+0 (3.5e-1) +
WFG5	3.15e-1 (1.3e-3)	3.16e-1 (1.4e-3) +	3.15e-1 (2.2e-3) ≈	2.75e-1 (1.1e-2) −	2.16e-1 (1.9e-3) −	2.16e-1 (1.4e-3) −	**2.13e-1 (3.8e-4) −**	1.29e+0 (1.5e-1) +	1.33e+0 (5.2e-1) +
WFG6	3.61e-1 (4.5e-3)	3.62e-1 (3.9e-3) ≈	3.60e-1 (2.4e-2) ≈	2.85e-1 (1.6e-2) −	**2.13e-1 (4.3e-3) −**	2.23e-1 (5.8e-3) −	2.16e-1 (4.7e-3) −	5.66e-1 (1.1e-1) +	1.38e+0 (1.0e-1) +
WFG7	3.29e-1 (3.3e-3)	3.29e-1 (4.2e-3) ≈	3.32e-1 (6.9e-3) +	2.62e-1 (1.5e-2) −	**2.03e-1 (8.7e-5) −**	2.07e-1 (2.5e-3) −	**2.03e-1 (2.2e-4) −**	1.12e+0 (2.1e-1) +	1.09e+0 (2.3e-1) +
WFG8	4.16e-1 (3.0e-2)	4.29e-1 (3.4e-2) ≈	4.19e-1 (2.0e-2) ≈	3.53e-1 (6.9e-3) −	**2.60e-1 (4.3e-3) −**	2.79e-1 (6.5e-3) −	2.61e-1 (5.2e-3) −	9.57e-1 (9.0e-2) +	1.64e+0 (1.5e-1) +
WFG9	3.44e-1 (1.8e-3)	3.43e-1 (3.9e-3) ≈	3.44e-1 (2.0e-3) ≈	3.04e-1 (2.0e-2) −	**2.03e-1 (4.5e-2) −**	2.10e-1 (4.2e-2) −	2.46e-1 (4.4e-2) −	5.36e-1 (8.7e-2) +	6.89e-1 (1.3e-1) +
+/≈/−		3/6/0	2/7/0	0/2/7	0/2/7	1/1/7	0/1/8	8/1/0	8/0/1

Median (interquartile range) over 30 runs; the best median on each problem is in bold; +, ≈ and − mark comparison algorithms that are significantly worse than, not significantly different from, or significantly better than MOEA/D-DoL (two-sided Wilcoxon rank-sum test of the 30 final values, Holm-corrected over the eight comparisons on each problem, α = 0.05; direction from the medians); reference fronts sampled as in PlatEMO, except that of WFG3, which is represented by 4667 points of its true front (Section 5.1).

## Appendix B

MOEA/D-DoL was tuned in three rounds of pilot runs with seeds 101–110, which no reported run uses, and with the budgets and population sizes of the reported runs (Table A4). The first round compared three stimulus settings of an earlier colony rule, in which a working worker always lowered its threshold and an idle one raised it, with three ablations, MOEA/D-DE and MOEA/D-FRRMAB; no colony setting beat MOEA/D-DE on any problem. A second round refined that rule, but it was stopped after 108 of 550 runs and its results were not kept; the rule was then replaced by the relative rule of Equation (18), and *θ*_max_ was raised from 1 to 4. The third round compared four settings of the relative rule. Relative to the setting that was kept, the other three had a 2–6% lower geometric-mean median IGD, so the choice rests on the rank rule and on the absence of a significant loss against MOEA/D-DE rather than on accuracy. The exponent *n*, the initial values, *β*, *θ*_min_, *s*_max_ and the MOEA/D-DE core were never varied in a pilot. The pilot runs were made before a floor of 10^−6^ was put on the components of the weight vectors, as PlatEMO does, so the code used for the reported runs reproduces their early generations exactly only with that floor removed, and over a full run, floating-point differences accumulate (final IGD within about 1.5% in the runs we repeated). The parameter study of Section 5.5, run with the code of the reported runs on six of the pilot problems, spans the values of *δ* and *ξ* compared in the last pilot and finds no significant effect of either.

**Table A4:** Pilot runs that fixed the colony parameters of MOEA/D-DoL.

Stage	Problems and Seeds	Settings Compared	Outcome
Pilot 1	UF1–UF4, UF7–UF9, DTLZ1, DTLZ3, DTLZ4, DTLZ7; seeds 101–105; results kept for 7 of the 11 problems	Earlier rule (threshold falls with any work and rises when idle), *θ* ∈ [0.05, 1]; (*δ*,*α*) = (0.05, 0.3), (0.10, 0.5), (0.02, 0.2); three ablations; MOEA/D-DE and MOEA/D-FRRMAB	Slowest setting best by mean rank of mean IGD (2.71 of 8; third by median IGD, 3.29, behind MOEA/D-DE and MOEA/D-FRRMAB at 3.14); no colony setting better than MOEA/D-DE on any problem
Pilot 2	Same 11 problems; seeds 101–105; stopped after 108 of 550 runs	Earlier rule around the winner of pilot 1: six (*δ*,*α*) and two (*ξ*,*ϕ*) settings and two ablations	Not kept; the rule was replaced by the relative rule of Equation (18) and *θ*_max_ raised from 1 to 4
Pilot 3	UF1–UF4, UF7–UF9, DTLZ4, DTLZ7; seeds 101–110	Relative rule, *θ* ∈ [0.05, 4], *α* = 0.2: (*δ*,*ξ*) = (0.10, 0.2), (0.05, 0.2), (0.10, 0.1), (0.20, 0.2); DoL-all, MOEA/D-DE and MOEA/D-FRRMAB	(0.10, 0.1) and (0.20, 0.2) share the best median-IGD rank (3.33 of 7); of the two, only (0.10, 0.1) has no significant loss against MOEA/D-DE and is kept; the other three settings have a 2–6% lower geometric-mean median IGD

Seeds 101–110 are disjoint from seeds 1–30 of the reported runs; budgets and population sizes are those of the reported runs. Tests are two-sided rank-sum tests at 0.05 without correction, as in the original pilots.

## Appendix C

Table A5 lists all eleven instruments of the cost-neutral schedule with the highest participation for the calibrations of Table 8, together with the highest effective marginal tax rate (EMTR) of each schedule, the combined marginal withdrawal of the benefit taper, the income tax and the phase-out of the credit, net of its phase-in, at any earnings for either household type. Table A6 reports the checks of Section 6.3 and Section 6.4. The capped problem adds to the baseline the constraint that this rate does not exceed 100%, imposed by a penalty that leaves no infeasible point in the final populations. The widened problem extends the ranges of Table 2 to *G* ∈ [0.05, 0.80], *g_L_* ∈ [0, 0.50], *d* ∈ [0, 1], *t_w_* ∈ [0.05, 1], *τ* ∈ [0.05, 0.80], *A* ∈ [0, 2], *r_in_* ∈ [0, 1], *K_max_* ∈ [0, 0.80], *y_po_* ∈ [0.10, 2.50] and *r_out_* ∈ [0.02, 1]; the range of *σ_cc_* is already [0, 1]. The joint draws vary the seven calibration parameters of Table 8 together by Latin hypercube sampling over the intervals spanned by their alternative values (for the two participation profiles, between the baseline and the alternative).

**Table A5:** Instruments of the cost-neutral schedule with the highest participation under the calibrations of Table 8.

Calibration	*G*	*g_L_*	*d*	*t_w_*	*τ*	*A*	*r_in_*	*K_max_*	*y_po_*	*r_out_*	*σ_cc_*	EMTR
Baseline	0.12	0.000	0.60	0.25	0.60	0.38	0.58	0.40	1.50	0.20	0.80	85%
*η_S_* = 0.10	0.10	0.000	0.59	0.21	0.60	0.55	0.59	0.36	1.20	0.44	0.12	104%
*η_S_* = 0.40	0.10	0.001	0.31	0.21	0.60	0.60	0.60	0.39	0.88	0.33	1.00	93%
*η_L_* = 0.25	0.11	0.022	0.58	0.20	0.60	0.75	0.60	0.40	0.29	0.28	0.64	108%
*η_L_* = 0.75	0.10	0.030	0.52	0.21	0.60	0.51	0.58	0.32	1.38	0.21	0.52	81%
*κ* = 0.10	0.10	0.027	0.51	0.22	0.60	0.44	0.60	0.40	1.30	0.45	0.91	104%
*κ* = 0.20	0.10	0.054	0.60	0.23	0.60	0.40	0.53	0.40	1.15	0.21	1.00	103%
*σ* = 0.4	0.10	0.000	0.60	0.20	0.60	0.50	0.57	0.40	0.75	0.49	0.94	129%
*σ* = 0.8	0.10	0.001	0.59	0.41	0.60	0.73	0.60	0.40	1.15	0.25	0.43	101%
*π_L_* = 0.06	0.11	0.006	0.60	0.20	0.59	0.64	0.60	0.35	0.61	0.32	0.96	111%
*π_L_* = 0.20	0.11	0.000	0.55	0.39	0.60	0.39	0.59	0.39	0.64	0.05	0.99	104%
$F_{S,i}^{0}$ 0.50–0.88	0.10	0.008	0.39	0.20	0.56	0.45	0.58	0.40	1.15	0.19	0.37	76%
$F_{L,i}^{0}$ 0.30–0.80	0.11	0.000	0.53	0.20	0.59	0.48	0.60	0.40	1.04	0.41	0.96	120%

Instruments as in Table 2; EMTR is the highest combined marginal withdrawal rate of the schedule over earnings, for either household type. Each row pools ten AR-MOEA runs.

**Table A6:** Robustness of the cost-neutral schedule with the highest participation to a marginal-rate cap, wider ranges and joint calibration draws.

Variant	Runs	Max *P* (%)	Gain (pp)	*G*	*g_L_*	*d*	*t_w_*	*τ*	*r_in_*	*K_max_*	*σ_cc_*	EMTR (%)
Table 2 ranges	30	91.4	19.2	0.11	0.00	0.59	0.20	0.59	0.60	0.39	0.03	92
Capped at 100%	30	91.7	19.5	0.10	0.00	0.60	0.20	0.59	0.51	0.39	0.90	95
Widened ranges	30	94.1	22.0	0.05	0.00	0.96	0.05	0.80	0.98	0.52	0.62	94
Widened and capped	30	94.0	21.8	0.05	0.00	0.69	0.05	0.73	0.99	0.80	0.19	85
Joint draws	50 × 10	88.0 (77.1–94.8)	18.5 (9.7–23.8)	0.11 (0.10–0.27)	0.00 (0.00–0.30)	0.58 (0.36–0.60)	0.21 (0.20–0.46)	0.60 (0.42–0.60)	0.60 (0.52–0.60)	0.39 (0.15–0.40)	0.82 (0.17–1.00)	98 (78–141)

Each row describes the schedule with the highest participation among those whose net cost is within 0.01 of the status quo’s; gain in percentage points over the status quo, computed from unrounded participation rates; EMTR as in Table A5. The first four rows pool 30 AR-MOEA runs; the joint draws give the median and, in parentheses, the range over 50 calibrations of ten runs each.
